# Multi-angle meta-analysis of the gut microbiome in Autism Spectrum Disorder: a step toward understanding patient subgroups

**DOI:** 10.1038/s41598-022-21327-9

**Published:** 2022-10-11

**Authors:** Kiana A. West, Xiaochen Yin, Erica M. Rutherford, Brendan Wee, Jinlyung Choi, Brianna S. Chrisman, Kaiti L. Dunlap, Roberta L. Hannibal, Wiputra Hartono, Michelle Lin, Edward Raack, Kayleen Sabino, Yonggan Wu, Dennis P. Wall, Maude M. David, Karim Dabbagh, Todd Z. DeSantis, Shoko Iwai

**Affiliations:** 1 Second Genome Inc., Brisbane, CA USA; 2 Department of Bioengineering, Stanford University, Stanford, CA USA; 3 Departments of Pediatrics (Systems Medicine), Stanford University, Stanford, CA USA; 4 Labii Inc., South San Francisco, CA USA; 5 Department of Biomedical Data Science, Stanford University, Stanford, CA USA; 6 Department of Psychiatry and Behavioral Sciences (By Courtesy), Stanford University, Stanford, CA USA; 7 Department of Microbiology, Oregon State University, Corvallis, OR USA; 8 Department of Pharmaceutical Sciences, Oregon State University, Corvallis, OR USA

**Keywords:** Autism spectrum disorders, Microbiome, Metagenomics

## Abstract

Observational studies have shown that the composition of the human gut microbiome in children diagnosed with Autism Spectrum Disorder (ASD) differs significantly from that of their neurotypical (NT) counterparts. Thus far, reported ASD-specific microbiome signatures have been inconsistent. To uncover reproducible signatures, we compiled 10 publicly available raw amplicon and metagenomic sequencing datasets alongside new data generated from an internal cohort (the largest ASD cohort to date), unified them with standardized pre-processing methods, and conducted a comprehensive meta-analysis of all taxa and variables detected across multiple studies. By screening metadata to test associations between the microbiome and 52 variables in multiple patient subsets and across multiple datasets, we determined that differentially abundant taxa in ASD versus NT children were dependent upon age, sex, and bowel function, thus marking these variables as potential confounders in case–control ASD studies. Several taxa, including the strains *Bacteroides stercoris* t__190463 and Clostridium M bolteae t__180407, and the species *Granulicatella elegans* and *Massilioclostridium coli*, exhibited differential abundance in ASD compared to NT children only after subjects with bowel dysfunction were removed. Adjusting for age, sex and bowel function resulted in adding or removing significantly differentially abundant taxa in ASD-diagnosed individuals, emphasizing the importance of collecting and controlling for these metadata. We have performed the largest (n = 690) and most comprehensive systematic analysis of ASD gut microbiome data to date. Our study demonstrated the importance of accounting for confounding variables when designing statistical comparative analyses of ASD- and NT-associated gut bacterial profiles. Mitigating these confounders identified robust microbial signatures across cohorts, signifying the importance of accounting for these factors in comparative analyses of ASD and NT-associated gut profiles. Such studies will advance the understanding of different patient groups to deliver appropriate therapeutics by identifying microbiome traits germane to the specific ASD phenotype.

## Introduction

The prevalence of Autism Spectrum Disorder (ASD) continues to rise^1^, and mounting evidence implies a potential role of the gut microbiome in ASD symptomatology. The core behavioral features of ASD are often accompanied by multiple comorbidities such as gastrointestinal (GI) and immune dysfunction^2^. A highly metabolically active entity, the gut microbiome resides at the intersection of numerous communication axes in the body^3^. Its involvement in immune development^4,5^, mood disorders^6^, and other extra-GI disorders has been established. Seemingly countless observational studies have found significant variability in the bacterial^7–20^ and fungal^19^ populations in ASD-diagnosed v. neurotypical children, but signals have not been robust across cohorts. Consistency in linking specific features or signatures to the ASD phenotype has proven futile to date^21–24^.

Due to the varying degree of behavioral symptoms and unique set of clinical features each individual will display, the ASD phenotype is extremely heterogeneous^25^. Between 40 and 70% of children with ASD experience GI abnormalities such as constipation and diarrhea^26,27^. While stool consistency has been shown to affect the observed microbial profile significantly^28,29^, this variable is not properly controlled in many studies. A recent reanalysis^30^ of a published dataset^19^ concluded that the original results were confounded by constipation in the ASD subjects. Additionally, human gut microbial populations fluctuate dramatically with age, particularly during early childhood^31^. ASD is typically diagnosed within the first 4 years of life^32^ yet there exists high variation in the median age of the past cohorts ranging from 3 to 11 years. This study explores variation in age of test subjects and mismatches in comorbidities between case and control groups, as they likely contribute to the inconsistencies observed across investigations conducted.

Leveraging previously published microbiome sequencing data from case–control studies, we identified relationships between specific variables and the gut microbiomes of all ASD children, as well as those observed only in subsets of the children studied. Utilizing linear mixed-effects models for meta-analysis, we identified bacterial taxa whose differential abundances were consistent across studies. We show that age, sex, and bowel function are important confounders that must be controlled and evaluated in a consistent manner when appraising inter-study ASD datasets. A discussion and synopsis of the sample sizes and statistical methods necessary to account for these variables and thus more accurately estimate ASD-associated effect size(s) ensues. The approach described here will aid in understanding the heterogeneity of the patient groups, leading to the identification of appropriate patient subsets to deliver targeted therapeutics in the future.

## Methods

### Dataset selection and inclusion

PubMed and the Sequence Read Archive (SRA; National Center for Biotechnology Information) were searched in March 2019 to identify publicly available raw datasets collected from case–control studies investigating the gut microbiota in ASD. The following queries yielded 72 potential publications/datasets: “autism[Title/Abstract] AND (microbiome[Title/Abstract] OR microbiota[Title/Abstract]) AND (16S OR sequencing OR metagenomic)” and “(autism gut) AND bioproject_sra[filter]” for PubMed and SRA, respectively. Inclusion criteria were adopted as follows: case–control ASD studies with human subjects under 18 years of age, datasets generated by 16S rRNA gene amplicon or metagenomic sequencing of human fecal samples, and raw data deposited in a public repository. Nine publications and 1 additional BioProject met all inclusion criteria, and an additional 6 publications met all criteria except for the availability of raw data (Supplementary file 1: Table S1). Of these 6, only 1 author provided raw data upon request. In total, 11 raw datasets associated with the 11 included studies were obtained. Three additional datasets acquired from an internal case–control ASD cohort^33^ were also included. One public dataset was subsequently excluded due to corrupted FASTQ files, bringing the final total to 13 datasets representing 10 cohorts. For datasets where repeated samples were collected, a single time point per individual was selected at random to include in the analysis.

### Metadata variable selection

Metadata were curated according to the Second Genome controlled vocabulary. Metadata variables which were present in multiple studies with values for n ≥ 6 individuals per group per study were included in the analysis. Those present in a single study were excluded. All possible pairing combinations of the included metadata variables were also used in the analysis to test each variable in subsets of subjects (Supplementary file 2: Fig. S1). In the combinations, each value of each categorical variable was used to subset subjects and test microbiota associations with the remaining variables. Original contrasts were labelled with the prefix “Subset: None” (e.g. Subset: None; Variable: Autism Spectrum Disorder—FALSE over TRUE) while contrasts performed in combination with another variable were labelled with the subset group value (e.g. Subset: Biological sex = Male; Variable: Autism Spectrum Disorder—FALSE over TRUE).

### Data processing

Raw data were downloaded from the respective repositories, obtained directly from authors (Supplementary file 1: Table S1), or generated in-house (see Supplementary file 3). Data were processed using standardized pipelines for each sequencing technology. For 16S rRNA gene amplicon sequencing data generated with Illumina technology, the DADA2 workflow was used with default settings for filtering, learning errors, dereplication, amplicon sequence variant (ASV) inference, and chimera removal^34^. Truncation quality was set to 2, and ten nucleotides were trimmed from each terminus of each read for both forward and reverse. Data generated using pyrosequencing technology, and any sequencing data with trimmed reads, were merged (for paired-end sequencing) then aligned to an in-house strain database (StrainSelect, strainselect.secondgenome.com, version 2019 i.e. SS19) as described in the next section. Remaining sequences without unique strain matches were quality filtered, dereplicated, and clustered (97%) with UPARSE to generate de-novo operational taxonomic units (OTUs). For metagenomic shotgun sequencing data, adapter sequences and low-quality ends were trimmed using Trimmomatic^35^ (< Q20), then contaminant sequences were removed using Bowtie2^36^. Host sequences were removed with Kraken^37^ and rRNA sequences were removed with SortMeRNA^38^. Sourmash^39^ was used to generate compressed representations of DNA sequences. Data generated using PhyloChip hybridization technology^40^ (see Supplementary file 3) were processed with the Sinfonietta software (Second Genome, Inc, Brisbane, CA) as previously described^41^, generating empirical OTUs (eOTUs).

### Strain-level annotations

Across all data types, consistently formatted identifiers for known strains were assigned using the SS19 database. SS19 contains known microbial strains publicized as of July 22, 2019. The abundance of each strain within shotgun metagenomic reads was calculated using Sourmash (kmer = 51, scaled = 5000). Using USEARCH^42^, ASVs matching a unique strain (≥ 99% global alignment identity) in the database were annotated with the strain identifier *only* if no genes from different strains had equivalent or higher identity matches than the unique strain hit. Abundances were summed for all ASVs matching the same strain. If a unique strain match was not achieved for an ASV, then species level and higher taxonomic placement was estimated with sintax (-cutoff 0.80)^43^. Genome Taxonomy Database (GTBD^44^) taxonomic nomenclature for species and higher taxa were used where available. Overall, 6%, 0.9%, and 6.8% of 16S ASVs, OTUs and eOTUs, respectively, were unique matches to strains.

### Statistical analysis

Within each dataset, taxonomic units (e.g. strains, sequence variants) present in less than 5% of biospecimens were removed. In addition, biospecimens with a sequencing depth less than 1% of the mean sequencing depth in the respective dataset were removed. To identify variables most associated with changes in bacterial community composition in each dataset, permutational multivariate analysis of variance, also known as Adonis (R package “vegan”) was performed for each variable/combination at each taxonomic rank.

For individual taxa, effect sizes (fold change in log_2_ scale) and standard errors were calculated within each dataset for each variable/combination at every taxonomic rank (phylum to species levels). Calculations for non-aggregated (strain level) 16S rRNA gene amplicon sequencing data were obtained using DESeq2^45^. Given that sourmash results in a table of relative abundances, effect sizes and standard errors were calculated using the escalc function (measure = “ROM”) in the “metaphor” R package^46^ to obtain log transformed ratios of means which were then transformed to log_2_ scale. Metagenomic data were not aggregated to higher taxonomic ranks. Aggregated 16S data were first transformed to relative abundance then effect sizes and standard errors were calculated as above. PhyloChip fluorescence intensity data were log_2_ transformed and here, escalc (measure = “MD”) was utilized to calculate the raw mean difference between groups which is equivalent to log_2_(fold change).

To identify taxa with concordant effect sizes for any given metadata variable/combination across multiple datasets, linear mixed-effects models^46^ were calculated using the rma.mv function in the “metafor” package. Each model was computed by regressing the effect sizes for a single taxon against a fixed effect, the metadata variable/combination, while controlling for the random variability introduced by each distinct dataset. The random-effects model can be written as *y*_*i*_ = *µ* + *u*_*i*_ + *ε*_*i*_, where *u*_*i*_* ∼ N*(0*, τ*^2^) and *ε*_*i*_* ∼ N*(0,* v*_*i*_). For a set of *i* = 1,..., *k* independent studies, *y*_*i*_ denotes the observed effect size in the *i*th study, *µ* denotes the average true effect, and *τ*^*2*^ is the variance in the true effects. Sampling variances are equal to *v*_*i*_, where *v*_*i*_ is the square of the standard errors of the estimates. In cases where multiple datasets originated from the same cohort study, inner (dataset) and outer (cohort) levels of the random effect were specified; datasets within a given cohort share correlated random effects. *P* values were adjusted according to the Benjamini–Hochberg method for all models within a given taxonomic rank. A floor of 1e-10 was applied to adjusted *P* values. Plots were generated using R packages “ggplot” and “ggpubr”. See Supplementary file 2: Fig. S2 for a schematic of the methods.

## Results

### Concordance of metadata variables across studies

Raw sequencing datasets (n = 10) were collected and re-analyzed from publicly available case–control studies. These ten datasets, representing nine distinct cohorts, were evaluated alongside three microbiome datasets from an internal cohort (Table 1). Datasets consisted of 16S rRNA gene amplicon and metagenomic DNA sequences. In addition, one internal dataset was generated using PhyloChip technology. Amplicon sequencing datasets covered multiple 16S rRNA variable regions. Cohorts included subjects between 2 and 18 years of age from the United States, Canada, China, Italy, and India. Participants were reported to be age-matched in 7 of the 10 studies although we found a significant difference in the ages of ASD v. NT children in one study^17^ (Supplementary file 2: Fig. S3). Strati et al.^19^ did not report the minimum and maximum ages of their participant pool.

**Table 1:** Summary of datasets.

Dataset	Publication	Technology	Sequencing instrument	16S region	ASD (n)	NT (n)	Minimum age (years)	Maximum age (years)	Median age (years)	Male (%)
DS1	Kang et al. 2013	16S HTS	454 Genome Sequencer FLX Titanium	V2V3	20	20	3	16	6	87.5
DS2	Kang et al. 2018	16S HTS	454 Genome Sequencer FLX Titanium	V2V3	23	21	4	17	9	84.1
DS3	Kang et al. 2017 Kang et al. 2019	16S HTS	Illumina MiSeq	V4	18	20	7.1	16.7	11.05	89.5
DS4	Li et al. 2019	16S HTS	Illumina HiSeq	V1V2	59	30	2	10	Not reported	Not reported
DS5	Strati et al. 2017	16S HTS	454 Genome Sequencer FLX	V3V5	39	39	Not reported	Not reported	Not reported	Not reported
DS6	Coretti et al. 2018	16S HTS	Illumina MiSeq	V3V4	11	14	2	4	Not reported	Not reported
DS7	Wang et al. 2019	MTG	Illumina HiSeq 4000	–	43	31	2	8	4	73.0
DS8	Pulikkan et al. 2018	16S HTS	Illumina NextSeq 500	V3	29	24	3	16	9	75.5
DS9	Averina et al. 2020	MTG	Illumina HiSeq 4000	–	29	20	2	9	3	79.6
DS10		16S HTS	Illumina MiSeq	V3V4	15	5	2	9	3.5	70.0
DS11	Internal	16S HTS	Illumina MiSeq	V4	107	92	2	11.83	4.75	72.4
DS12		PhyloChip	Thermo-Fisher Gene Titan	V1V9	96	93	2	11.83	5.17	72.0
DS13		MTG	Illumina NextSeq	–	96	91	2	11.83	5.17	73.3

*HTS* high-throughput sequencing, *MTG* metagenomics.

**Figure 1:**
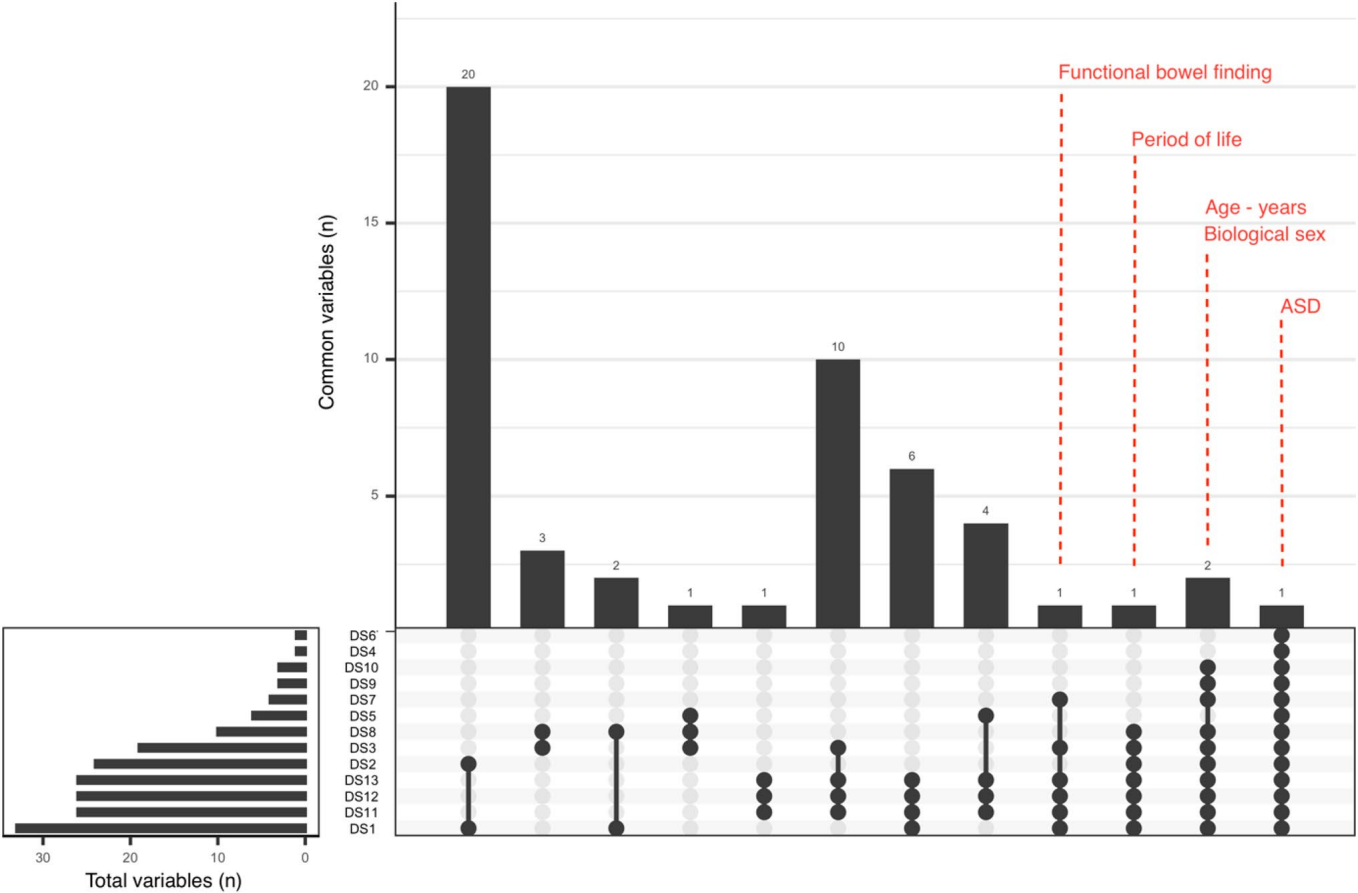
Concordance of metadata variables across datasets. Each bar represents the number of common variables between datasets and solid black circles below the bar indicate datasets that contain the intersected set of variables. The “Total variables” inset displays the total number of metadata variables for each dataset. The top (present in at least 6 datasets) intersected variables are labelled while the remaining variables are listed in Supplementary file 1: Table S2.

To identify microbial features associated with specific metadata variables across datasets, we first examined all the variables that were concordant across studies. A total of 52 distinct variables were reported (Supplementary file 1: Table S2). Age (10 of 13 datasets), sex (10 of 13 datasets), and bowel function (6 of 13 datasets) were among the most prevalent metadata categories. Of the 52 variables selected for meta-analysis, the vast majority were present in fewer than five datasets, and only five variables were present in more than four datasets (Fig. 1). To investigate the impact of potential confounders, all possible pairwise combinations of variables were also included in the meta-analysis, resulting in 580 tests (Fig. 1b).

### Multi-cohort bacterial abundance signatures germane to ASD exist at all taxonomic ranks

**Figure 2:**
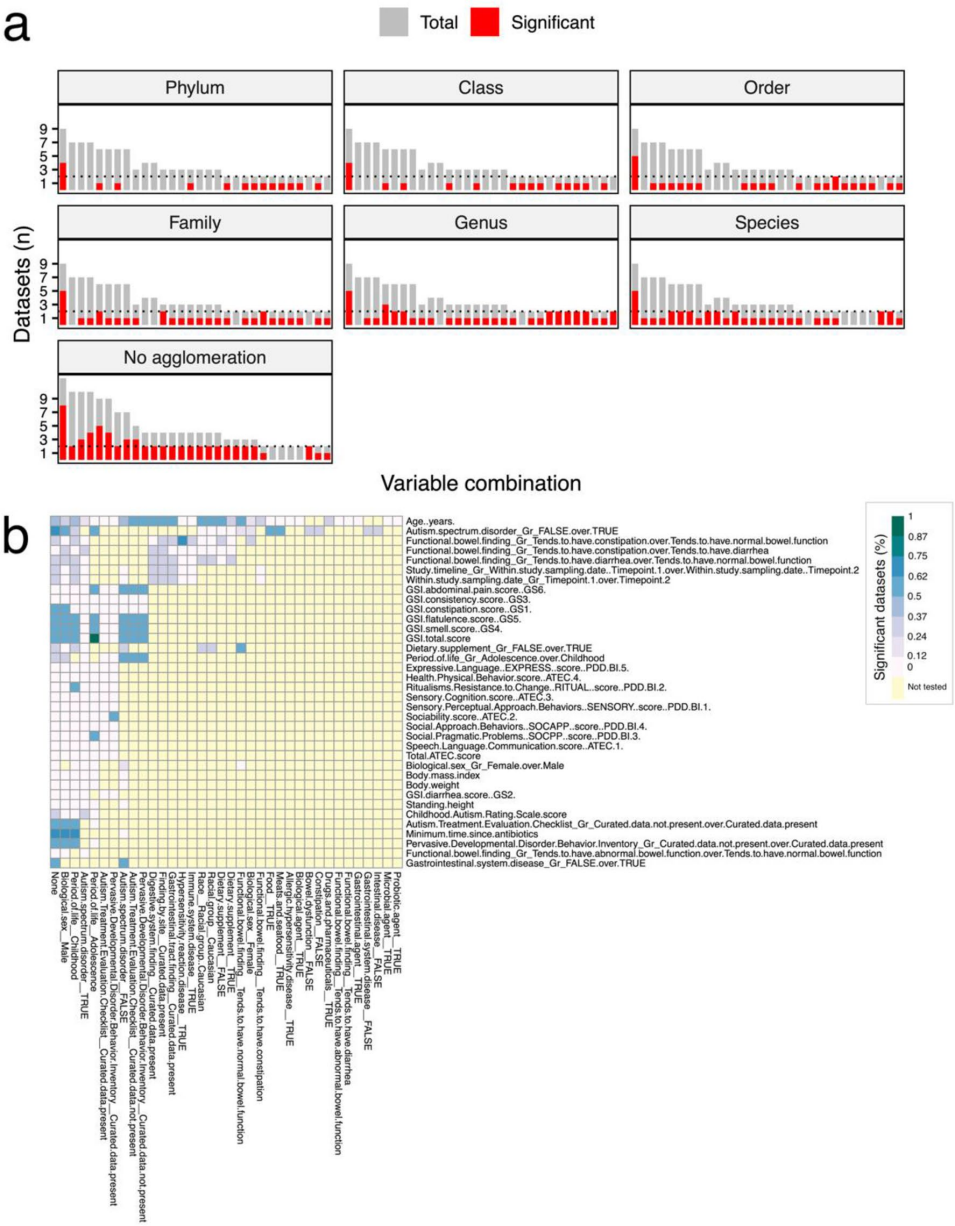
Variables associated with beta-diversity in multiple datasets. (**a**) Variables/combinations significantly associated with changes in bacterial community structure in at least 2 datasets are plotted. Each bar represents the total number of datasets in which a variable (or variable combination) was tested and the red fraction of the bar denotes the number of datasets where the difference in beta-diversity was significant (PERMANOVA, *P* < 0.05). The first bar in each panel represents the variable “Subset: None; Autism Spectrum Disorder—FALSE over TRUE.” Remaining variables are listed in Supplementary file 1: Table S3. (**b**) Proportions of datasets with significant tests (non-aggregated data) for each variable combination are plotted. Columns are used to arrange subset values while rows segregate test variables. Results from aggregated data (genus and species levels) are presented in Supplementary file 2 (Figs. S4, S5).

To assess the association between bacterial community structure (beta-diversity) and each of the metadata variables/variable combinations, Adonis tests were performed on non-aggregated data (i.e. ASV or OTU level) as well as data aggregated at each taxonomic rank. Non-aggregated data yielded the greatest number of significant (*P* < 0.05) tests (Fig. 2a). The main broad variable under investigation, denoted as “Subset: None; Autism Spectrum Disorder—FALSE over TRUE”, was significant in several datasets at every taxonomic rank. Of the 580 possible combinatorial metadata tests, 30 were significantly associated with bacterial community structure in at least two datasets and at least one taxonomic rank (Supplementary file 1: Table S3). Thirteen of the 30 tests focused on differences in subject age. Age was a significant contributor to bacterial community variation within the NT population (“Subset: Autism Spectrum Disorder = FALSE”) in four out of nine datasets, but was only significant in two of 10 datasets in the ASD population (“Subset: Autism Spectrum Disorder = TRUE”). Significant changes in beta-diversity for each variable within each subset are shown in Fig. 2b. Variables bearing the greatest number of dataset associations were found in healthy, male children (Subsets: “Biological sex = Male,” “Period of life = Childhood,” and “Autism Spectrum Disorder = FALSE”).

**Figure 3:**
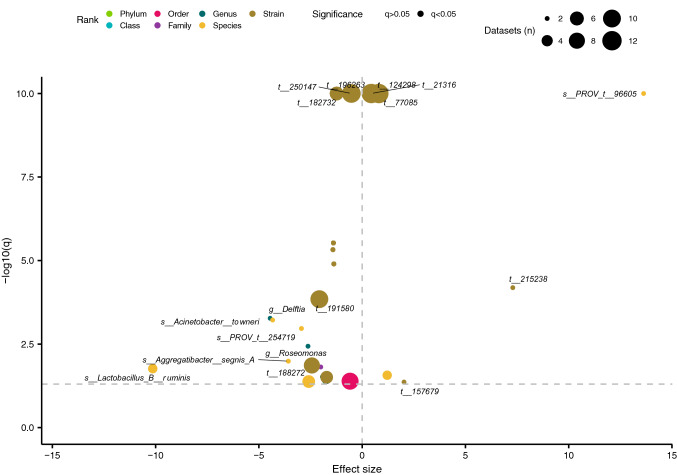
Features associated with ASD at different taxonomic levels. Effect sizes and *q*-values from random-effects models (meta-analysis) are plotted. Each model represents an association between the abundance of a specific taxon and ASD (negative direction) or NT (positive direction). The color, transparency, and size of each point denotes the taxonomic rank of the taxon, the significance of the model, and the number of datasets included in the model, respectively. Horizontal dotted line indicates significance threshold (q = 0.05). Significant models are labelled by the taxon investigated. Strain-level results are reported in Table S4.

In addition to community-wide associations, relationships between individual taxa and each variable/variable combination were assessed using random-effects models. When comparing ASD and NT children (“Subset: None; Autism Spectrum Disorder—FALSE over TRUE”; Fig. 3) with aggregated data, *Burkholderiales* (order level) was detected in nine of the 10 datasets (metagenomic data were not aggregated) and was significantly more abundant in children with ASD, although the effect size was small (log_2_(fold change) = − 0.59). An unannotated species, *s__PROV_t__172009* (genus *Lawsonibacter*), was also enriched in the ASD cohorts (present in five of the 10 datasets). While remaining meta-analysis results from aggregated data were weak, with significant taxa detected in only three datasets, non-aggregated data revealed more consistent associations. Two sequence variants, annotated as unclassified strains of the species *Ruminiclostridium_E siraeum*, were less abundant in ASD cohorts and detected in 12 of 13 datasets (Supplementary file 1: Table S4). *Barnesiella intestinihominis* and *Faecalibacterium prausnitzii_K* were also depleted in the ASD cohorts from 11 and 9 datasets, respectively. Taxa most differentially abundant in the ASD-diagnosed cohorts and exhibiting the largest effect sizes included: *Fusicatenibacter saccharivorans* (10 datasets), *Bacteroides uniformis* (8 datasets), and *Bacteroides thetaiotaomicron* (5 datasets).

### Heterogeneity of the ASD phenotype is evident in taxonomic profiles affected by GI dysfunction

**Figure 4:**
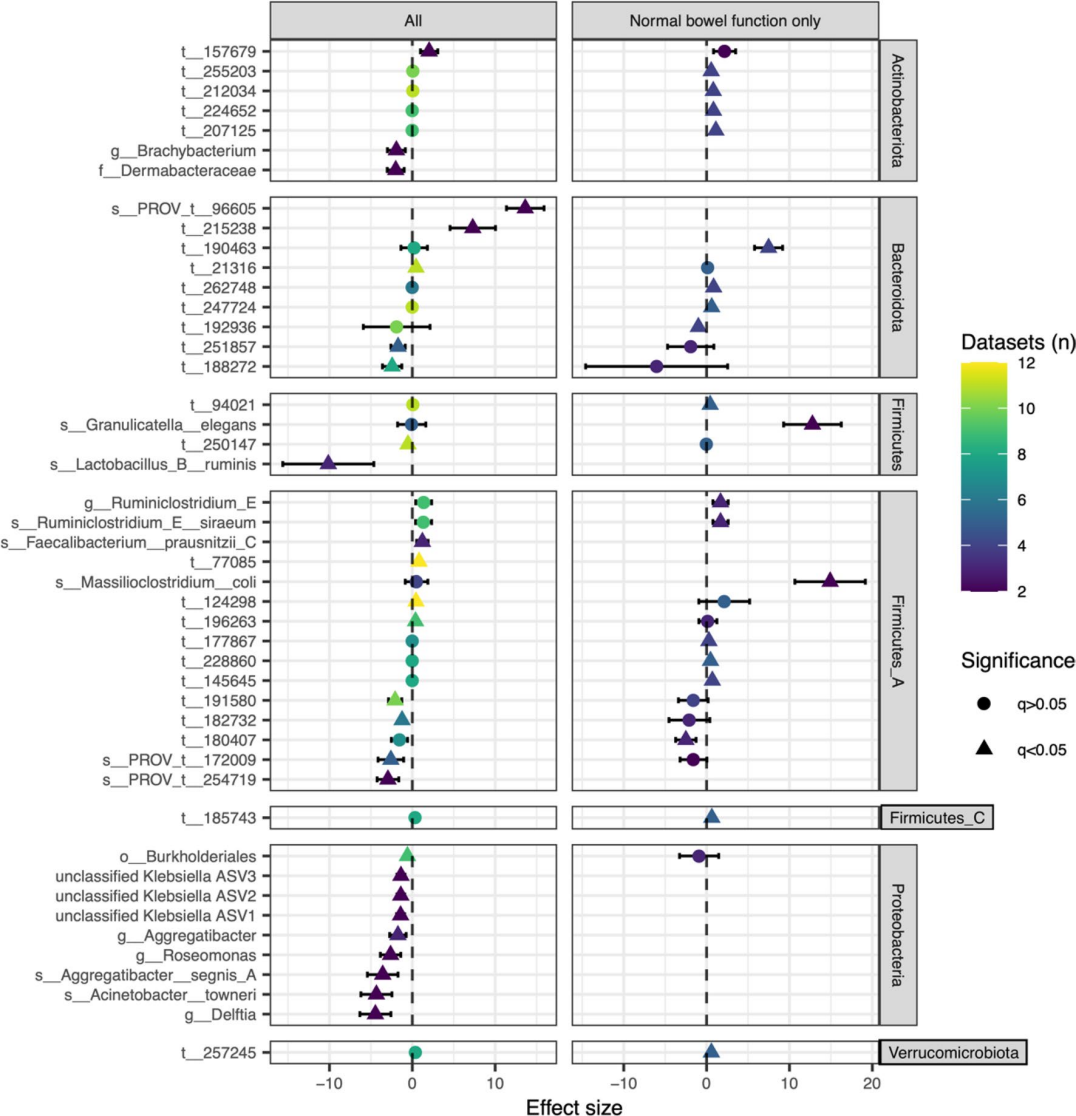
Bowel dysfunction confounds relationships linking ASD to differential bacterial taxa abundances. Taxa significantly associated with NT (positive direction) or ASD (negative direction) by meta-analysis are plotted by phylum. Taxonomic label prefixes indicate the taxon’s rank where t__, s__, g__ or f__ are strain, species, genus or family, respectively. ASD-associated signatures are different when children with bowel dysfunction are included (left panels) compared to when only children with normal bowel function are considered (right panels). Missing data points indicate taxa absent from the remaining datasets and individuals. Effect sizes and significance were calculated using random-effects models.

We next explored the differential taxonomic findings in subsets of patients. Due to the influence of GI function on gut bacterial profiles^47,48^ and the high prevalence of GI dysfunction in the ASD population^26,49^, we elected to appraise ASD-associated differential taxa abundances in children without bowel dysfunction (“Subset: Tends to have normal bowel function; Autism Spectrum Disorder—FALSE over TRUE”). Removing children with bowel dysfunction invalidated many significant ASD v. NT findings suggesting the initial findings were more related to bowel function than study group, although the number of datasets and participants that could be investigated in the subset was reduced (Fig. 4, Supplementary file 2: Fig. S3). Several bacterial taxa exhibited differential abundance in ASD v. NT children only after subjects with bowel dysfunction were removed, indicating a close relationship with ASD diagnosis rather than constipation/diarrhea. As expected, the gut bacterial community profiles of ASD individuals with constipation differed greatly from those of ASD individuals with diarrhea (Supplementary file 2: Fig. S6), which confounds the case–control contrast. Many taxa discriminated between ASD subjects afflicted with constipation and those afflicted with diarrhea, including members of the *Lachnospiraceae* family and species of *Bacteroides, Bifidobacterium,* and *Streptococcus* (Supplementary file 1: Table S5). The number of differentially abundant taxa was far greater than that observed when comparing ASD and NT children (Supplementary file 1: Table S4), suggesting that bowel dysfunction exerts a greater influence on microbial community dynamics than the ASD phenotype.

Although the effect sizes of most ASD-associated taxa in children with normal bowel function were small, 19 significant taxa (*q* < 0.05) were identified. We discuss the top four (based on effect size): *Massilioclostridium coli* (species, depleted in ASD), *Granulicatella elegans* (species, depleted in ASD), t__190463 (*Bacteroides stercoris* strain, depleted in ASD), and t__180407 (*Clostridium M bolteae* strain, enriched in ASD). The two species, *M. coli* and *G. elegans*, were completely absent from ASD patients, but had low prevalence in the NT tested (7% and 3%, respectively; 2 datasets). The two strains, t__190463 and t__180407, were prevalent (25% and 38%, respectively) and detected in 4 and 3 datasets, respectively. Effect sizes from individual studies in addition to pooled effect sizes are reported in Supplementary file 2: Fig. S7. Changes in sample size due to the removal of children with bowel dysfunction are reported in Supplementary file 2: Fig. S8.

### Age and sex confound comparative analyses of bacterial differential abundance in ASD v. NT children

**Figure 5:**
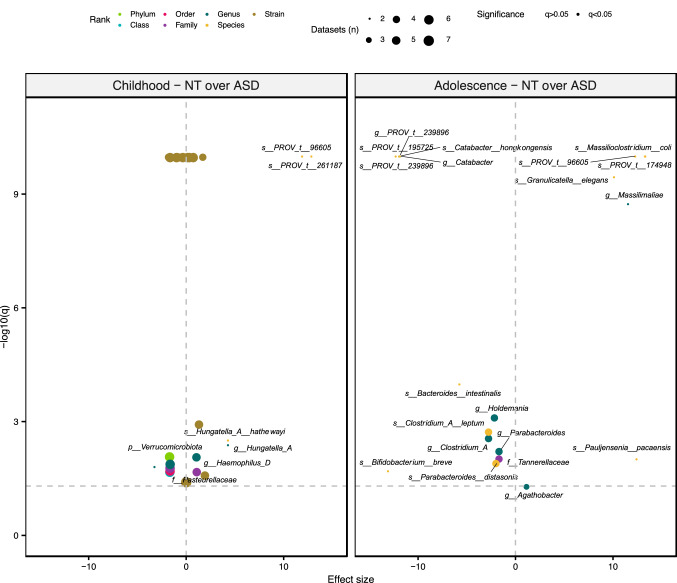
ASD-associated taxa abundance depends on the period of life studied. Effect sizes and *q*-values from random-effects models investigating the associations between different taxa and NT (positive direction) or ASD (negative direction) at different stages of childhood are plotted. The color, transparency, and size of each point denotes the taxonomic rank of the taxon, the significance of the model, and the number of datasets included in the model, respectively. Horizontal dotted line indicates significance threshold (q = 0.05). Significant models are labelled by the taxon investigated except for strain-level results. These models are reported in Supplementary file 1: Table S6.

Evidence suggests that the gut microbiome continues to evolve and mature well into childhood^31^. Given the wide age range of children with ASD studied, it is likely that some inconsistencies in the literature stem from age-related development of the gut microbiota. In this study, two distinct periods of life were considered: childhood, i.e., 2–9 years old, and adolescence, i.e., 10–17 years old. The abundances of several bacterial taxa differed between ASD and NT subjects only when considering children, or only when considering adolescents (Fig. 5). Though only detected in two datasets, one unannotated species (*s__PROV_t__96605*, genus *Prevotella*) was significantly depleted in the ASD groups, irrespective of age.

**Figure 6:**
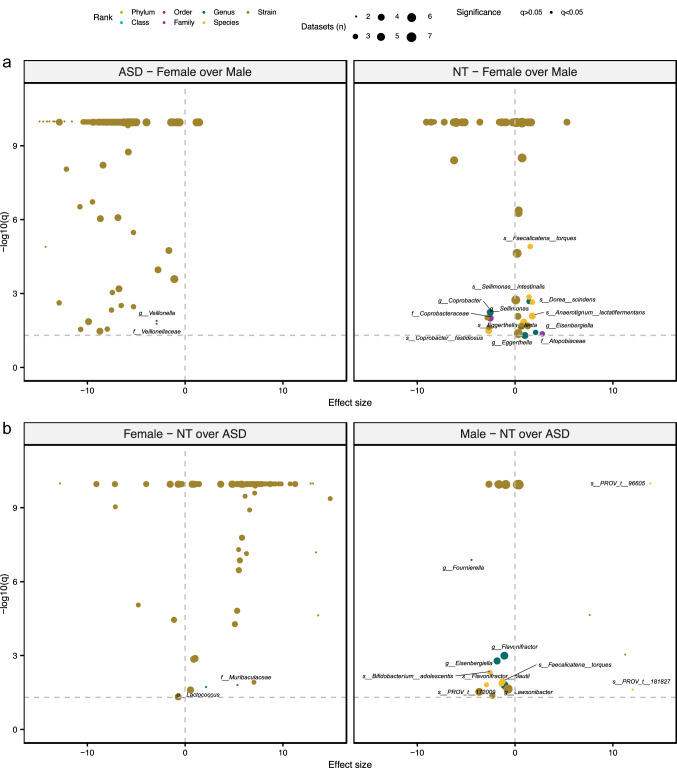
Sex-dependent differences in ASD v. NT populations. (**a**) Effect sizes and *q*-values from random-effects models investigating the associations between different taxa and sex (female: positive direction, male: negative direction) in the ASD compared to NT groups are plotted. (**b**) Models investigating ASD-associated taxa (NT: positive direction, ASD: negative direction) in either males or females are also plotted. The color, transparency, and size of each point denotes the taxonomic rank of the taxon, the significance of the model, and the number of datasets included in the model, respectively. Horizontal dotted line indicates significance threshold (q = 0.05). Significant models are labelled by the taxon investigated except for strain-level results. These models are reported in Supplementary file 1: Table S7.

Sex bias plays a significant role in ASD studies due to the elevated prevalence (~ 4X) of diagnoses in males v. females^50^. While differences in gut bacterial composition have been documented between male and female subjects^51^, this confounder is often omitted from statistical analyses of ASD-associated microbial population flux. In this study we see that sex-dependent differences in the fecal microbiome are stronger in the ASD-diagnosed cohort. Taxa that discriminated between males and females were also specific to each study group (Fig. 6a). Only three taxa were significantly differentially abundant in both male and female ASD and NT children (Supplementary file 1: Table S7). Furthermore, there was no overlap in differentially abundant taxa between only male or only female ASD v. NT comparisons (Fig. 6b). The abundances of members of the *Lachnospiraceae* family and several species of *Bacteroides* and *Bifidobacterium* were elevated in the gut microbiomes of ASD-diagnosed males compared to their female counterparts. The abundance of these same taxa varied significantly between ASD children with constipation v. diarrhea (Supplementary file 1: Table S5). This hints at a potential interaction or shared pathway between the two confounders (sex and bowel function) in the ASD population.

### Adjusting for confounding factors eliminates noise in ASD-associated microbiome changes

Since we observed confounding for age, sex, and bowel function in microbiome changes, ASD-associated strains were evaluated in two datasets, i.e., DS1 and DS11, wherein all three metadata were reported across both ASD and NT study groups. In DS1, the abundances of four distinct strains were significantly different in ASD subjects when the confounders were omitted from consideration. With confounder adjustment, only one of the strains retained significance but seventeen additional strains were significantly differentially abundant (Supplementary file 2: Fig. S9). Initially, no differentially abundant strains were detected in DS11. After adjusting for confounders, however, a single strain was significantly more abundant in the ASD cohort (Supplementary file 2: Fig. S9).

## Discussion

Although there is convincing evidence that gut microbiome profiles differ between ASD and NT children, there is little consensus as to which bacterial taxa are impactful and/or at all relevant to ASD symptomatology. Independent studies yield results that are not generalizable to the ASD population, potentially due to the heterogeneity of the ASD phenotype and variability in age of subjects studied. Recent efforts to systematically review initiatives linking the microbiome to ASD and/or conduct meta-analyses on the reported results of such efforts show little to no empirically derived associations between varying microbiome diversity and the ASD phenotype^21–24^. We hypothesized that standardized pre-processing of raw data generated from ASD microbiome surveys coupled with downstream comprehensive meta-analyses would yield more insightful and more accurate findings. In a similar vein, we thoroughly investigated microbiome populations alongside all the metadata variables compiled from numerous studies to reconsider some differential findings in subsets of ASD v. NT children. We found that certain bacterial abundances were significantly different between ASD and NT, oftentimes the direct consequence(s) of the confounding effects of bowel dysfunction, age, and sex. Our statistical approach is simple, and results are directly interpretable, making it favorable for mining microbiome data.

Of the clinical comorbidities documented in children with ASD, GI issues are among the most common^52^. Diarrhea, constipation, and abdominal pain are the most frequently reported GI symptoms in the ASD population^53,54^, but many have alternating constipation/diarrhea so these categories are often inadequate to describe their GI symptoms over time. However, stool consistency (a proxy for constipation/diarrhea) has been reported as the top fecal microbiome covariate^28^ and has been shown to confound case–control studies^29^. Unsurprisingly, the works discussed here suggest that many of the differential taxa abundances previously thought to associate directly with the ASD phenotype were likely an artifact linked more closely to constipation and/or diarrhea in the subjects sampled. Our results align with a recent reanalysis^30^ of a dataset included here^19^. Only two previously published investigations^9,17^ and our internal cohort reported information relating to bowel function in both case and control groups. Given the significant impact of stool consistency on fecal microbial populations and the prevalence of diarrhea and constipation in the ASD population, current and future studies must consider, at minimum, scoring collected samples based on the Bristol Stool Scale^55^ and reporting this covariate index in statistical analyses.

Upon considering only individuals with normal bowel function, we were able to identify taxa more closely associated with ASD status, a feat not possible in isolated cohorts due to inadequate statistical power. Of note, three taxa were depleted in individuals with ASD compared to NT controls across multiple datasets: *Bacteroides stercoris* t__190463 (strain level), *Granulicatella elegans* (species level), and *Massilioclostridium coli* (species level). Although the two species of interest had low prevalence in the children studied, they were not detected in the ASD patients. *Bacteroides stercoris* was previously reported to have lower relative abundance in ASD compared to NT children^56^, but higher relative abundance in a different study^15^. Here, meta-analysis estimated reduced relative abundance in the ASD population after pooling signals from four different datasets, thus demonstrating its utility in uncovering democratized associations. Although evidence to suggest a potential mechanism for the depletion of *B. stercoris* in ASD pathology has yet to be demonstrated, we believe the strain identified warrants further investigation as a therapeutic. Meta-analysis also revealed that *Clostridium_M bolteae* t__180407 (strain level) was enriched in the ASD populations investigated (3 datasets). *Clostridium bolteae* has been investigated in the context of ASD and is consistently more abundant in these children compared to their NT counterparts^10,57,58^. Abundances are even higher in individuals with Pitt Hopkins syndrome^59^, a severe ASD with a high incidence of GI dysfunction. *C. bolteae* produces a conserved specific capsular polysaccharide which is immunogenic in rabbits and has been the focus of ASD vaccine efforts^60,61^. Although these findings are promising, more evidence is needed as relatively few datasets supported the result in this study and the strain level findings may be confounded by between-study heterogeneity (Supplementary file 2: Fig. S7).

Age is another covariate of immense importance and relevance to microbiome health and status, particularly in young children. The gut microbiome begins to resemble that of an adult sometime around age three^62^, but evidence suggests further maturation over the course of later childhood^31^. Studies on ASD have inadvertently targeted child subjects spanning a broad age range, and unfortunately age-dependent gut microbiome differences within each study group likely affect results and confound inter-study inferences. Seven of the 10 studies revisited herein ensured that case and control groups were age-matched, though none mentioned controlling for age in statistical analyses even though children of preschool age (2–4 years old) and teenagers (13–17 years old) were evaluated in the same study. A recent analysis of more than 2500 individuals revealed that disease-microbiome associations depend on the age group studied, and that adjusting for age improves the detection of microbes truly relevant to the disease phenotype^63^. Although demonstrated in adults, it is conceivable that this paradigm applies to children and adolescents who are growing and maturing rapidly. Our differential findings in young children v. adolescents support the notion that the microbiome differs according to age in non-adult populations as well.

Our findings demonstrate that ASD-associated bacterial taxa abundances differ innately as a function of sex. Due to the roughly four-times greater prevalence of ASD diagnoses in males v. females, study populations are often biased and imbalanced with respect to sex. Male subjects made up 70–89.5% of the cohorts examined here, indicating that sex-dependent microbiome associations were challenging to assess in the isolated studies prior. By exploiting meta-analytical approaches, we were able to show that sex exerts an even greater influence on the microbiome in children with ASD than those with typical development. In addition, associations between fecal microbiomes and ASD were stronger in females compared to males. Consistent with our findings (Adonis test), previous studies have reported no associations between gut microbial community structure and sex in healthy children^64–66^. However, we detected several taxa that were differentially abundant between male and female NT children, and these disparities were more drastic in male v. female ASD children, but only three strains were significant in both contrasts. There was no overlap in differentially abundant taxa between ASD and NT children in male and female subsets. These findings suggest that recent surveys of ASD-microbiome variation, all of which are based predominantly on male subjects, may not be generalizable to the female population.

Finally, the limited sample size of most studies including but not limited to those considered in this comprehensive analysis (n < 100 across nine cohorts; n < 50 across 5 cohorts) drastically restricts statistical power and thus experimental resolution. Considering only the extent of variability observed within the ASD population and the number of confounders that need to be addressed, it is overly apparent that larger studies are warranted to improve statistical power and strengthen downstream inferences and conclusions. The findings presented here strongly suggest that surveys investigating simple case–control contrasts are not suitable when investigating relationships between gut microbiome perturbations and the ASD phenotype. This work underscores the dire need to systematically collect, curate, and report highly detailed metadata. It is also apparent that statistical methods used to estimate effect sizes between cases and controls should integrate confounder adjustments to more accurately account for age, sex, and stool sample consistency, at a minimum. Other confounders not addressed by our analysis due to inadequate reporting include diet and medications. Dietary preference and medication usage are strong gut microbiome covariates^28,29^ and are particularly relevant to studies of ASD where case patients often have extreme food selectivity^67^ and medical comorbidities requiring pharmacological treatment^68^.

Our study demonstrates that the gut microbiomes of the ASD population exhibit appreciable heterogeneity, an observation that has been established regarding the clinical manifestations of the disorder. High within-group variability produces artifacts and masks true ASD-microbiome relationships. As population-scale studies of ASD may be difficult to establish, we demonstrated meta-analytical approaches with confounder adjustment to unveil gut bacterial disturbances directly related to ASD symptomatology. This is a substantial breakthrough in understanding the patient population and associated comorbidities, which will help lead to personalized microbiome-based therapeutics.

## Supplementary Information

Supplementary Information 1.

Supplementary Information 2.

Supplementary Information 3.

Supplementary Information 4.

## Data Availability

Accession numbers for publicly available raw data are detailed in Supplementary file 1: Table S1. Raw sequencing data generated from the internal cohort has been deposited at http://files.cgrb.oregonstate.edu/David_Lab/M3_longitudinal_16s/. Raw PhyloChip data generated from the internal cohort is available in MIAME format at https://greengenes.secondgenome.com/?prefix=downloads/phylochip_datasets/ (SG_SIwai_2021_M3_ASD_CIMA.tgz). All code used to generate the figures presented can be found in Supplementary file 4.

## Abbreviations

ASD: Autism Spectrum Disorder
ASV: Amplicon sequence variant
DNA: Deoxyribonucleic acid
eOTU: Empirical operational taxonomic unit
GI: Gastrointestinal
NT: Neurotypical
OTU: Operational taxonomic unit
rRNA: Ribosomal ribonucleic acid
SRA: Sequence Read Archive
