# Supplementary material for: Multi-angle meta-analysis of the gut microbiome in Autism Spectrum Disorder: a step toward understanding patient subgroups
Source: Sci Rep. 2022 Oct 11;12:17034. doi: 10.1038/s41598-022-21327-9 (PMC9554176; doi:10.1038/s41598-022-21327-9)
Supplement: Supplementary file 2 — Supplementary Information 2. [file 41598_2022_21327_MOESM2_ESM.pdf]

Supplementary Figures

Multi-angle meta-analysis of the gut microbiome in Autism Spectrum Disorder: a step toward understanding patient subgroups

Authors: Kiana A. West[1], Xiaochen Yin[1], Erica M. Rutherford[1], Brendan Wee[1], Jinlyung Choi[1], Brianna S. Chrisman[2], Kaiti L. Dunlap[3], Roberta L. Hannibal[1], Wiputra Hartono[1], Michelle Lin[1], Edward Raack[1], Kayleen Sabino[1], Yonggan Wu[1,4], Dennis P. Wall[3,5,6], Maude M. David[7,8], Karim Dabbagh[1], Todd Z. DeSantis[1], and Shoko Iwai[1]

Affiliations:
[1]Second Genome Inc., Brisbane, CA, United States
[2]Department of Bioengineering, Stanford University, Stanford, CA
[3]Departments of Pediatrics (Systems Medicine), Stanford University, Stanford, CA
[4]Labii Inc., South San Francisco, CA, United States
[5]Department of Biomedical Data Science, Stanford University, Stanford, CA
[6]Department of Psychiatry and Behavioral Sciences (by courtesy), Stanford University, Stanford, CA
[7]Oregon State University, Department of Microbiology, Corvallis, OR
[8]Oregon State University, Department of Pharmaceutical Sciences, Corvallis, OR

Metadata

Bowel function (BF)	Age	Gender	Medication
Diarrhea	6	Male	Yes
Constipation	4	Male	NA
Normal	7	Female	NA
Normal	3	Male	NA
Constipation	9	Male	NA
Diarrhea	7	Female	NA

Tests
Subset: None -> test Normal vs Diarrhea
Subset: None -> test Normal vs Constipation
Subset: None -> test Constipation vs Diarrhea
Subset: None -> test Age
Subset: None -> test Male vs Female
Subset: BF = Normal -> test Age
Subset: BF = Normal -> test Male vs Female
Subset: BF = Constipation -> test Age
Subset: BF = Constipation -> test Male vs Female
Subset: BF = Diarrhea -> test Age
Subset: BF = Diarrhea -> test Male vs Female
Subset: Gender = Male -> test Age
Subset: Gender = Male -> test Normal vs Diarrhea
Subset: Gender = Male -> test Normal vs Constipation
Subset: Gender = Male -> test Constipation vs Diarrhea
Subset: Gender = Female -> test Age
Subset: Gender = Female -> test Normal vs Diarrhea
Subset: Gender = Female -> test Normal vs Constipation
Subset: Gender = Female -> test Constipation vs Diarrhea

Figure S1: Example of variable combinations. For each dataset, metadata variables available for 6 or more subjects were evaluated. Remaining non-numeric variables with more than 2 categories were expanded to all possible 2-group contrasts and tested (Adonis and feature selection). Each value from each categorical variable was then used to identify subsets of subjects and all tests were repeated within each subset.

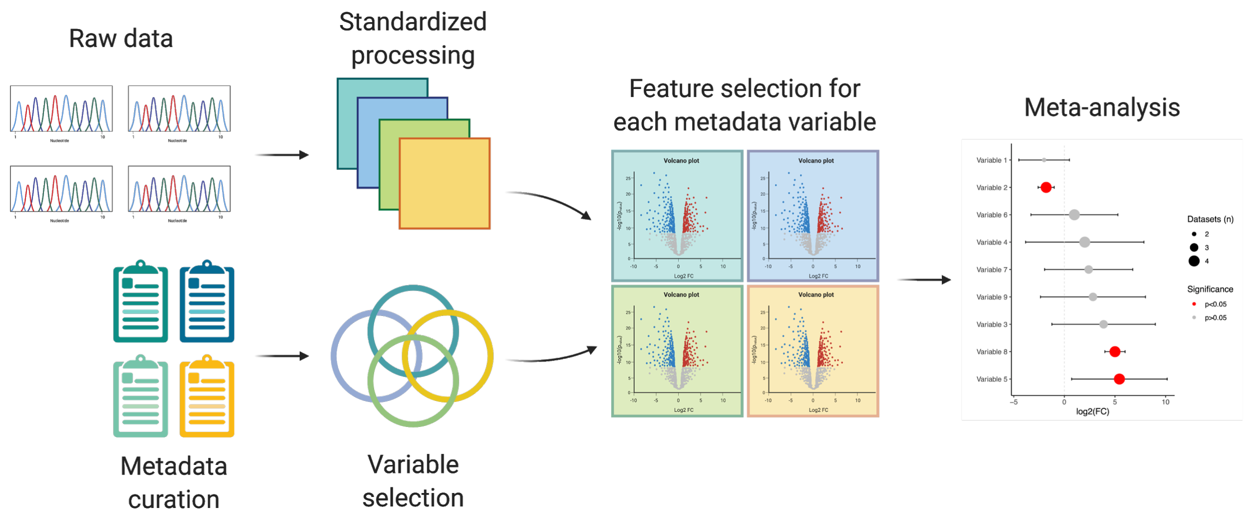

Figure S2: Data analysis workflow. Raw data were downloaded and preprocessed using standardized in-house pipelines. Metadata were curated according to the Second Genome controlled vocabulary and metadata variables were selected for inclusion in the analysis based on their prevalence in multiple studies. Combinations of variables were also generated (**see Supplementary Figure S1**). Feature selection was performed in each dataset individually for all included metadata variables and variable combinations. Meta-analysis was performed using effect sizes and standard errors for each feature (taxon)-variable pair across multiple datasets. *Figure generated with Biorender.*

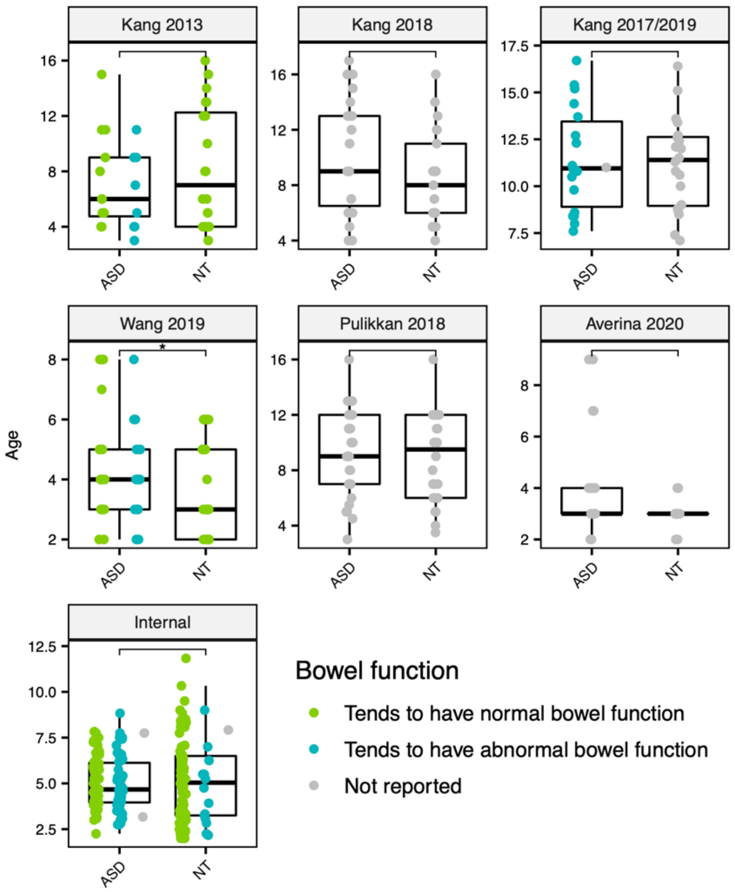

Figure S3: Age distribution by study group. For studies which reported ages of participants, age distributions are compared between study groups. Statistically significant differences in age between ASD and NT children are shown (*P < 0.05, Wilcoxon test). Another major confounder, bowel function, is also shown for each participant. Subsetting the ASD group to only children with normal bowel function reduces the number of studies and participants that can be included in the analysis. Likewise, there are not enough NT children with abnormal bowel function to run a meta-analysis.

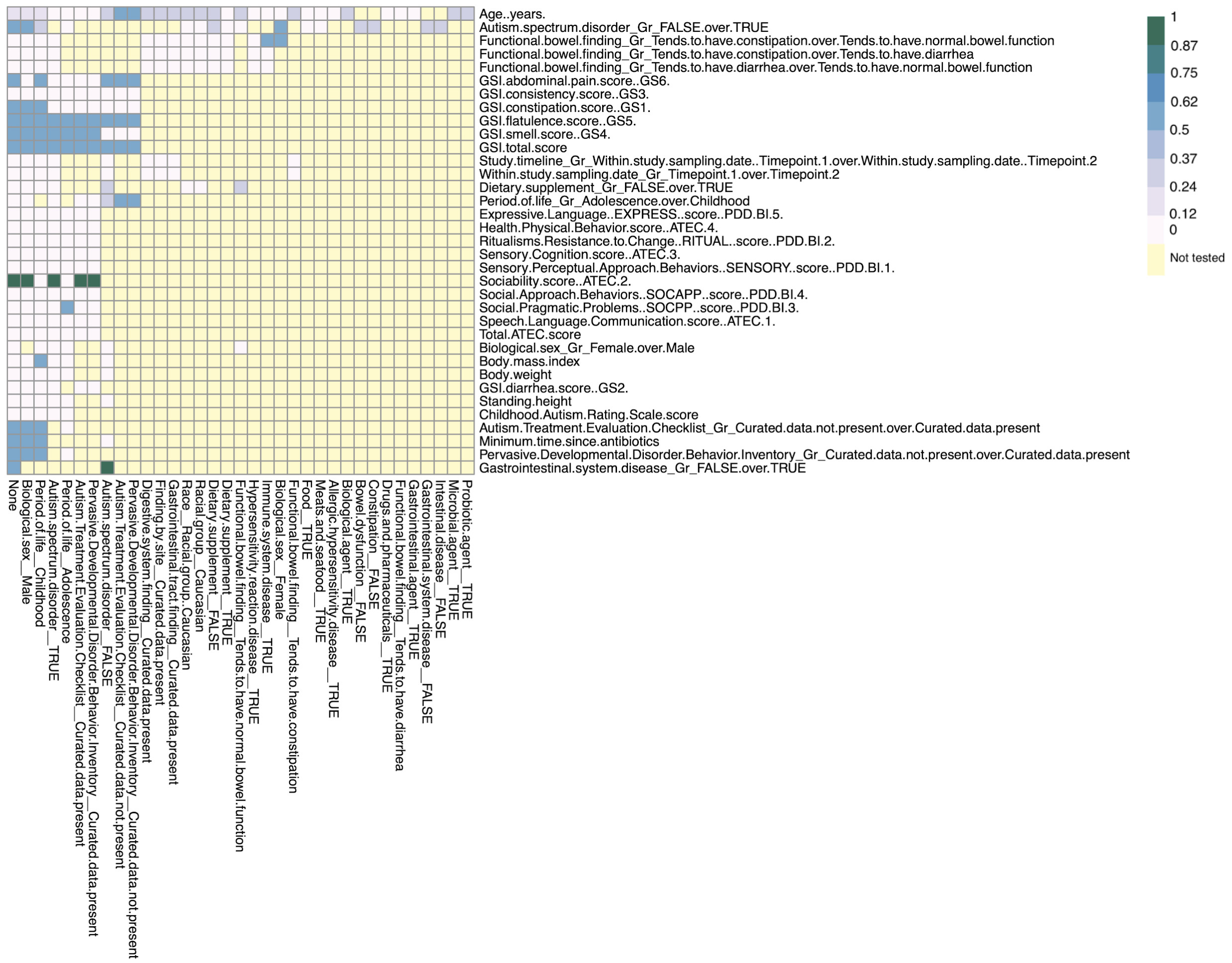

Figure S4: Variables associated with the gut bacterial community in subsets of individuals at genus level. Proportions of datasets with significant tests for each variable combination are plotted. Columns represent subset values while rows represent test variables.

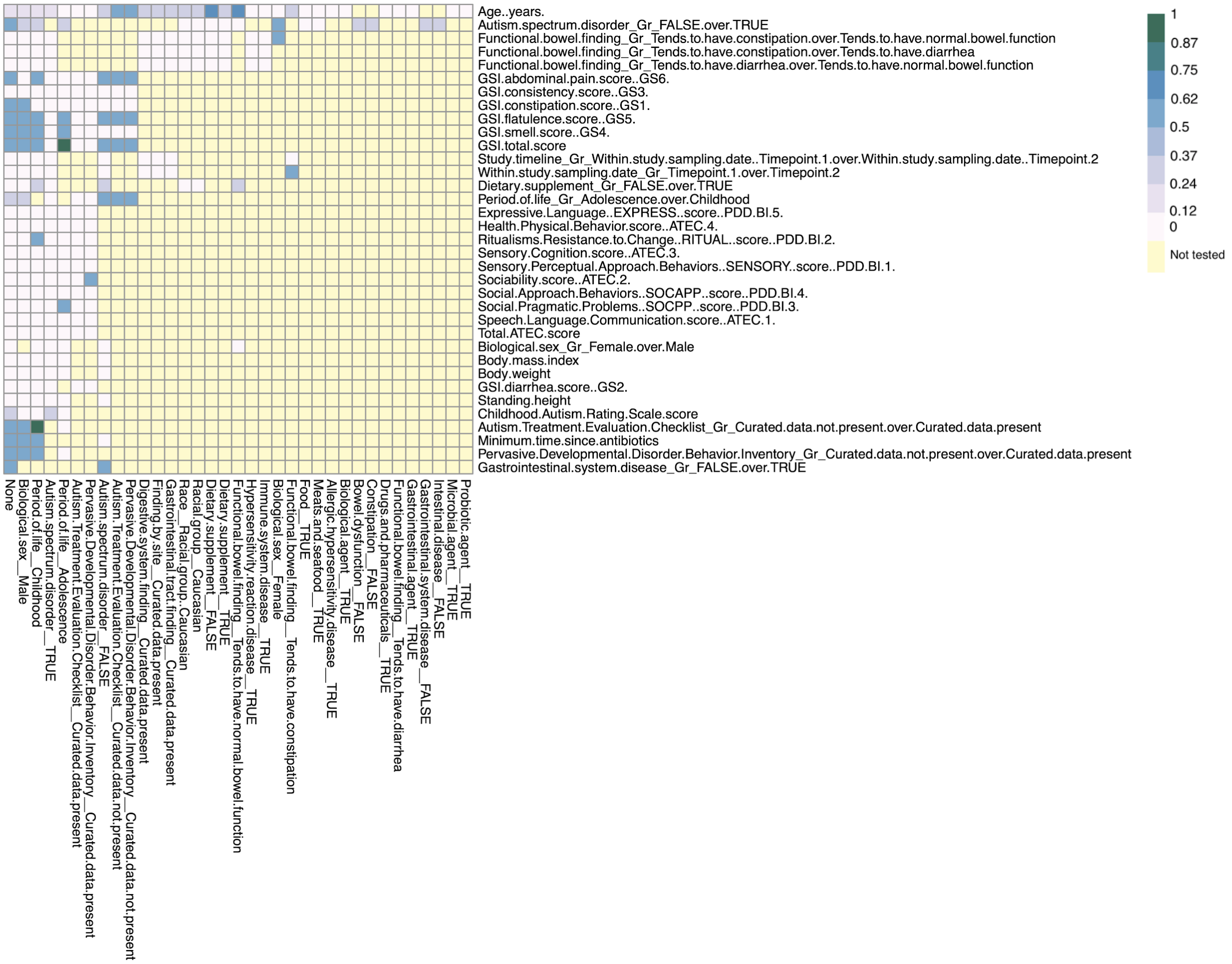

Figure S5: Variables associated with the gut bacterial community in subsets of individuals at species level. Proportions of datasets with significant tests for each variable combination are plotted. Columns represent subset values while rows represent test variables.

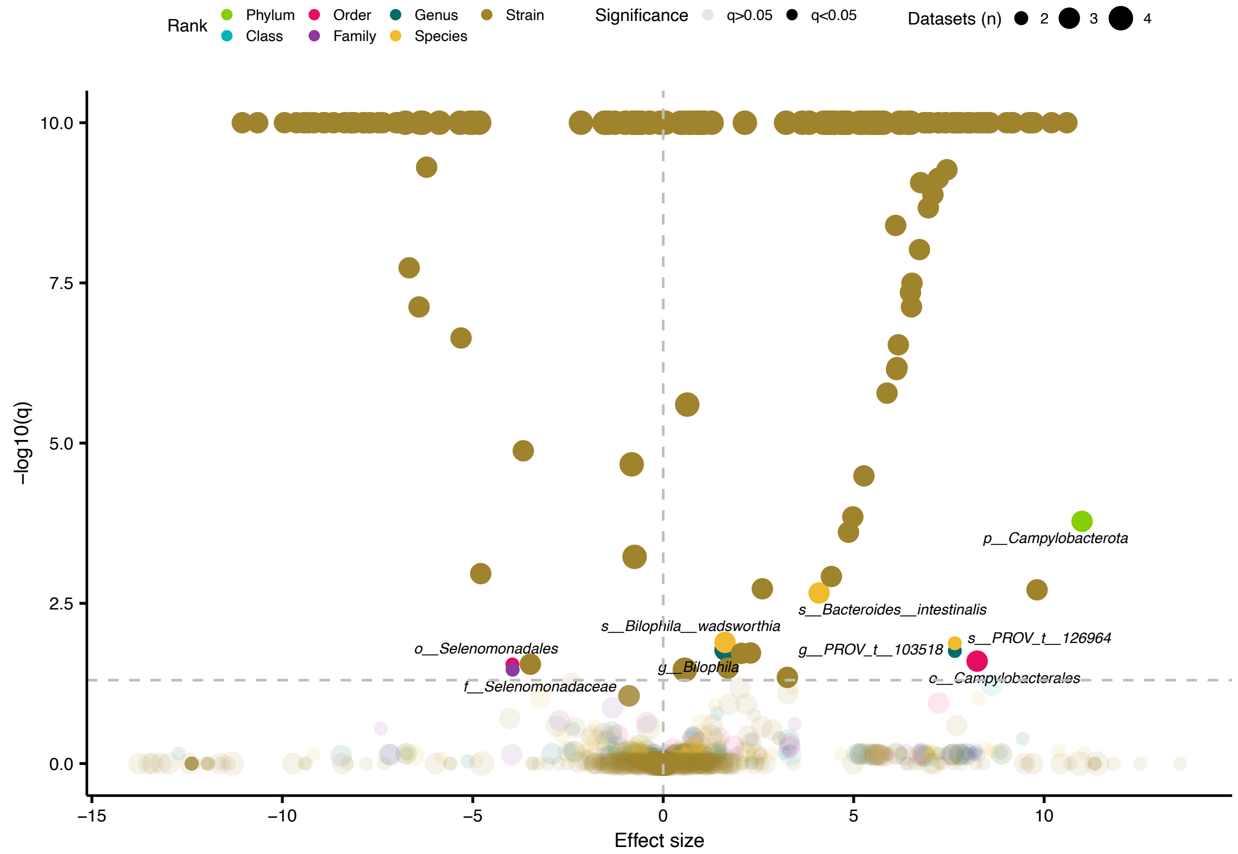

Figure S6: Bowel function significantly impacts gut bacterial profiles in children with ASD. Effect sizes and *q*-values from random-effects models testing associations between different taxa and constipation (positive direction) or diarrhea (negative direction) in ASD children are plotted. The color, transparency, and size of each point denotes the taxonomic rank of the taxon, the significance of the model, and the number of datasets included in the model, respectively. Horizontal dotted line indicates significance threshold (q = 0.05). Significant models are labelled by the taxon investigated except for strain-level results. These models are reported in **Additional file 1: Table S5**.

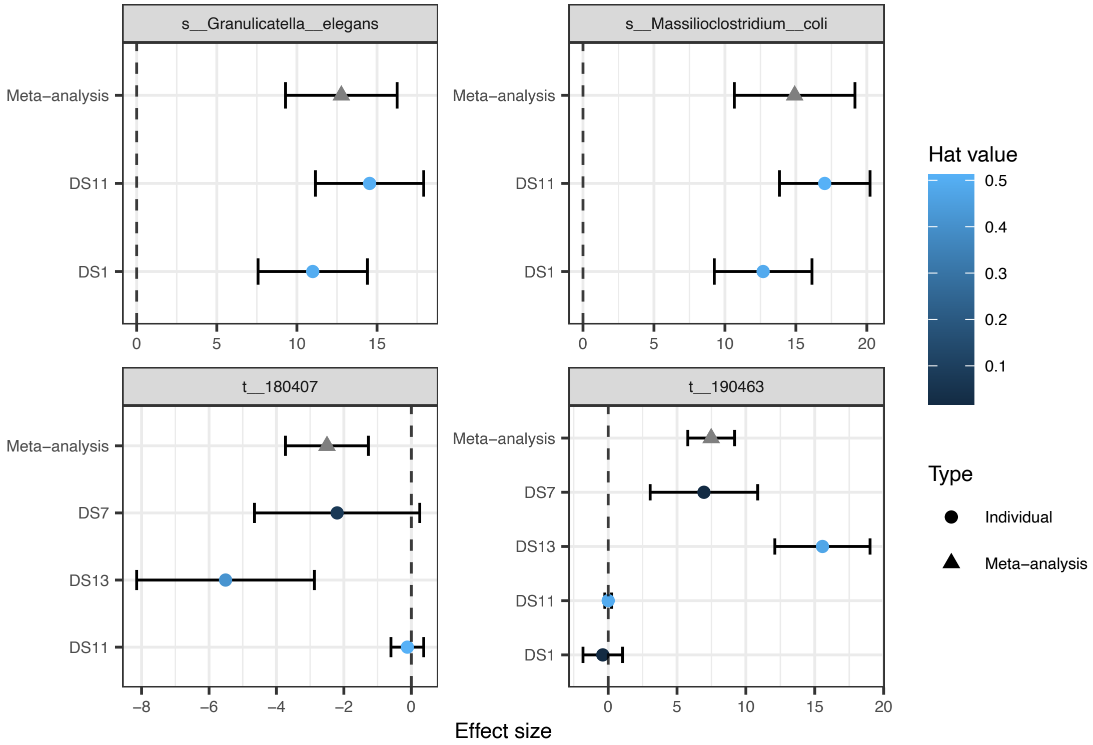

Figure S7: Individual and pooled effect sizes for differentially abundant taxa. After removing children with abnormal bowel function, four taxa were significantly (q < 0.05; |Effect size| > 2) differentially abundant in ASD (negative direction) compared to NT (positive direction) children. Meta-analyses represent pooled effect sizes calculated using random-effects models. Individual effect sizes from the contributing datasets are also plotted. Hat values represent the influence of each dataset in the model.

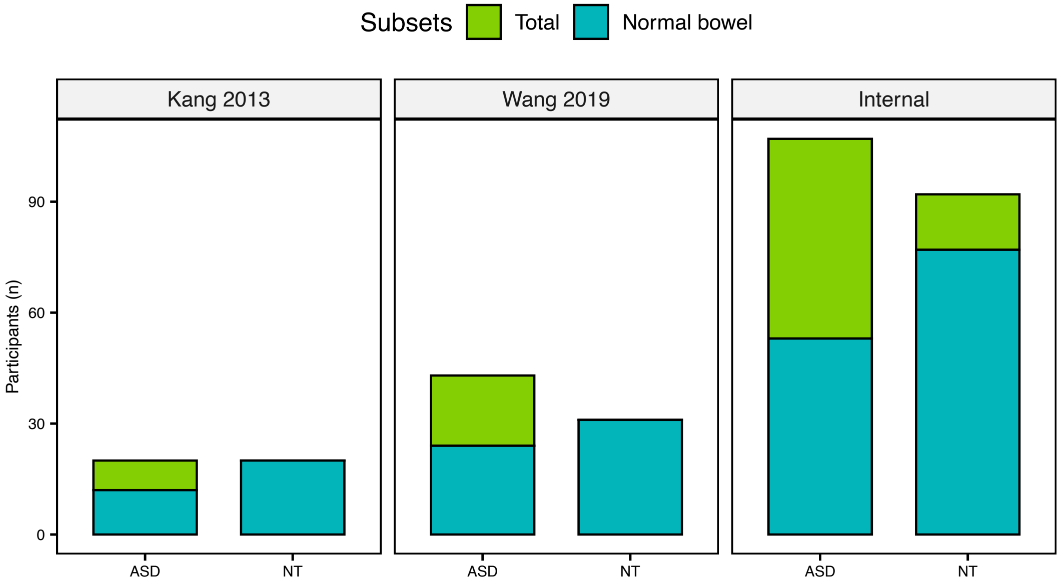

Figure S8: Changes in sample size due to the exclusion of children with abnormal bowel function. Above (Fig. S7), random-effects models were calculated to determine which taxa were differentially abundant in ASD v. NT children after removing those with abnormal bowel function. Changes in sample size for each study are reported here. Studies Kang 2013, Wang 2019, and Internal represent datasets DS1, DS7, and DS11/13, respectively.

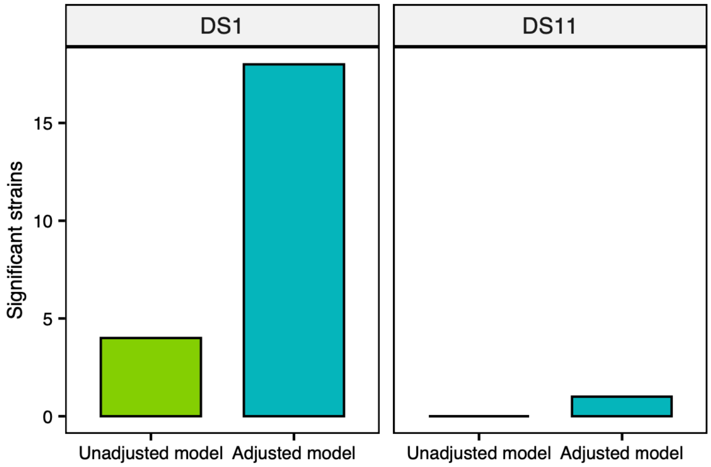

Figure S9: Confounder-adjusted feature selection detects additional ASD-associated strains. Differential abundance was evaluated using DESeq2 to identify strain-annotated sequence variants enriched or depleted in children diagnosed with ASD. The number of significant ($q < 0.05$) strains was compared between the unadjusted model (~Autism spectrum disorder) and the adjusted model (~Biological sex + Age + Functional bowel finding + Autism spectrum disorder).
