# Supplementary material for: Multi-angle meta-analysis of the gut microbiome in Autism Spectrum Disorder: a step toward understanding patient subgroups
Source: Sci Rep. 2022 Oct 11;12:17034. doi: 10.1038/s41598-022-21327-9 (PMC9554176; doi:10.1038/s41598-022-21327-9)
Supplement: Supplementary file 3 — Supplementary Information 3. [file 41598_2022_21327_MOESM3_ESM.pdf]

Additional file 3: Supplemental Methods

Multi-angle meta-analysis of the gut microbiome in Autism Spectrum Disorder: a step toward understanding patient subgroups

Authors: Kiana A. West[1], Xiaochen Yin[1], Erica M. Rutherford[1], Brendan Wee[1], Jinlyung Choi[1], Brianna S. Chrisman[2], Kaiti L. Dunlap[3], Roberta L. Hannibal[1], Wiputra Hartono[1], Michelle Lin[1], Edward Raack[1], Kayleen Sabino[1], Yonggan Wu[1,4], Dennis P. Wall[3,5,6], Maude M. David[7,8], Karim Dabbagh[1], Todd Z. DeSantis[1], and Shoko Iwai[1]

Affiliations:
[1]Second Genome Inc., Brisbane, CA, United States
[2]Department of Bioengineering, Stanford University, Stanford, CA
[3]Departments of Pediatrics (Systems Medicine), Stanford University, Stanford, CA
[4]Labii Inc., South San Francisco, CA, United States
[5]Department of Biomedical Data Science, Stanford University, Stanford, CA
[6]Department of Psychiatry and Behavioral Sciences (by courtesy), Stanford University, Stanford, CA
[7]Oregon State University, Department of Microbiology, Corvallis, OR
[8]Oregon State University, Department of Pharmaceutical Sciences, Corvallis, OR

Internal study cohort and biospecimen collection

Datasets 11, 12, and 13 included in the present meta-analysis were generated from an internal case-control cohort study[1]. Paired individuals were included based on the following criteria: two children (age 2-8 years) born +/-2 years apart, one child with an Autism Spectrum Disorder (ASD) diagnosis, and one child with no ASD diagnosis (neurotypical; NT). Each participant completed a standardized survey to collect information relating to race, age, bowel function, and other information relevant to the gut microbiome (e.g. medications and diet). Three stool samples (week 0, week 1, and week 2) were collected from each individual in preservative-enriched collection kits. All collected biospecimens were mailed overnight on ice to Second Genome and stored at -80C.

Biospecimen sample processing

Nucleic acid was extracted from each stool sample using the Qiagen MagAttract PowerMicrobiome DNA/RNA Kit according to manufacturer's guidelines and optimized for high-throughput processing. DNA concentrations were measured using the Qubit® Quant-iT dsDNA High Sensitivity Kit (Invitrogen, Life Technologies, Grand Island, NY). Extracted DNA was

subjected to amplicon sequencing, PhyloChip analysis, and metagenomic sequencing as described below.

16S rRNA gene amplicon sequencing

The V4 region of the 16S rRNA gene was amplified by PCR utilizing fusion primers designed against the surrounding conserved regions and tailed with sequences to incorporate Illumina (San Diego, CA) adapters and indexing barcodes. Each sample was PCR amplified with two differently bar coded V4 fusion primers[2]; PCR products were quantified by fluorometric method (Qubit or PicoGreen from Invitrogen, Life Technologies, Grand Island, NY). Amplicon samples were pooled in equimolar concentrations and sequenced on the MiSeq® platform for 250 cycles and with custom primers designed for paired-end sequencing. To minimize the biases of batch effect, six samples collected from a given family were sequenced in one batch alongside technical controls. Samples with fewer than 20,000 quality-filtered reads were re-sequenced up to three times.

PhyloChip 16S rRNA microarray analysis

PhyloChip, an Affymetrix GeneChip microarray developed at Lawrence Berkeley National Laboratory, uses signature probes (n=974,540) targeting all known 16S rRNA gene sequences (V1-9)[3] to quantify abundances of bacterial and archaeal taxa. Full-length 16S rRNA gene amplicons were generated by PCR (27F: 5'-AGRGTTTGATCMTGGCTCAG-3'; 1492R: 5'-GGTTACCTTGTTACGACTT-3') and concentrated using a solid-phase reversible immobilization method for the purification of PCR products. DNA was quantified using a Qubit® Quant-iT dsDNA Broad-Range Kit. For each sample, 500 ng were used with the addition of PhyloChip Control MixTM. Amplicons were then fragmented, biotin labeled, and hybridized to the PhyloChipTM Array (version G4). PhyloChip arrays were washed, stained, and scanned using a GeneTitan® scanner (Affymetrix). Each scan was captured using standard Affymetrix software (GeneChip® Microarray Analysis Suite).

Metagenomic sequencing

DNA samples were prepared using the Illumina NexteraXT kit and quantified with the Quant-iT dsDNA High Sensitivity assay. Libraries were pooled and sequenced on the Illumina NextSeq 550 platform (150 bp paired-end reads).

References

1. Chrisman BS, Paskov KM, Stockham N, Jung J-Y, Varma M, Washington PY, et al. Improved detection of disease-associated gut microbes using 16S sequence-based biomarkers. BMC Bioinformatics. 2021;22:509. doi:10.1186/s12859-021-04427-7.

2. Caporaso JG, Lauber CL, Walters WA, Berg-Lyons D, Huntley J, Fierer N, et al. Ultra-high-throughput microbial community analysis on the Illumina HiSeq and MiSeq platforms. ISME J. 2012;6:1621–4. doi:10.1038/ismej.2012.8.

3. Schatz MC, Phillippy AM, Gajer P, DeSantis TZ, Andersen GL, Ravel J. Integrated microbial survey analysis of prokaryotic communities for the PhyloChip microarray. Appl Environ Microbiol. 2010;76:5636–8. doi:10.1128/AEM.00303-10.
