# Supplementary material for: Multi-angle meta-analysis of the gut microbiome in Autism Spectrum Disorder: a step toward understanding patient subgroups
Source: Sci Rep. 2022 Oct 11;12:17034. doi: 10.1038/s41598-022-21327-9 (PMC9554176; doi:10.1038/s41598-022-21327-9)
Supplement: Supplementary file 4 — Supplementary Information 4. [file 41598_2022_21327_MOESM4_ESM.html]

Multi-angle meta-analysis of the gut microbiome in Autism Spectrum Disorder: a step toward understanding patient subgroups


# Multi-angle meta-analysis of the gut microbiome in Autism Spectrum Disorder: a step toward understanding patient subgroups

#### Kiana A. West

#### 2022-09-08

- Summary of datasets and
  metadata
  - Number
    of subjects (and their ages) from each dataset included in the
    analyses
    - Summary of
      cohort characteristics by study group
    - How
      many individuals remain after subsetting to normal bowel function
      only?
  - Common
    metadata variables across datasets (upset plot)
  - Number of tests
    after variable combinations
  - Percentage of
    strain hits in each 16S dataset
- Summary of Adonis results
  - Significance by variable
    combinations
- ASD-associated taxa
  - After
    subsetting
    - Add individual
      effect sizes for selected strains
    - Prevalence
      of ASD-associated taxa in children with normal bowel function
    - Within ASD group
      differences by bowel function
    - Childhood versus
      adolescence
    - Male vs
      Female
- ASD-associated
  taxa after adjusting for confounders

All code to generates figures for the manuscript is contained
here.

Download and read in strain taxonomy

```
system(paste("aws s3 cp", params$ss_tax, "taxonomy_of_strains.txt"))
ss_tax <- read_delim("taxonomy_of_strains.txt", delim = "\t")[,-1] # remove domain column
```

Read in results

```
ASD_datasets <- read_csv(params$datasets_included)
comparisons_all <- read_csv(params$comparisons_all_file)
comparisons_sub <- read_csv(params$comparisons_sub_file)
adonis_res <- read_csv(params$adonis_res_file)
fs_res <- data.table::fread(params$fs_res_file)
ma_res <- NULL

# add taxonomy to meta-analysis results
for (i in 1:6) {
  rank <- colnames(ss_tax)[i]
  x_tax <- ss_tax %>%
    dplyr::group_by_(rank) %>%
    summarise_at(.vars = vars(-c("StrainSelectID", "placement")), function(x) {paste0(unique(x), collapse = ";")})
  # for ranks lower than i, change to NA (except species level - lowest)
  if (i != 6) {
    x_tax[,colnames(ss_tax)[(i+1):6]] <- NA
  }
  # read in results
  x <- read_csv(paste0(params$ma_res_path, "meta_analysis_chao_Stool_bybin_", str_to_title(rank), ".csv")) %>%
    mutate(Rank = str_to_title(rank)) %>%
    # add taxonomy
    left_join(x_tax[,c("phylum", "class", "order", "family", "genus", "species")], by = c("Bin" = rank), keep = TRUE)
  ma_res <- bind_rows(ma_res, x)
}
# add taxonomy to strain-level results
x <- read_csv(paste0(params$ma_res_path, "meta_analysis_chao_Stool_bybin_NoAgglomeration.csv")) %>%
    mutate(Rank = "NoAgglomeration") %>%
    # add taxonomy
    left_join(ss_tax[,c("phylum", "class", "order", "family", "genus", "species", "StrainSelectID")], by = c("Bin"="StrainSelectID"))
ma_res <- bind_rows(ma_res, x)

# add floor for pvalues
ma_res$Padjust <- ifelse(ma_res$Padjust <= 1e-10, 1e-10, ma_res$Padjust)
```

Read in data

```
for (i in paste0("DS", 1:13)) {
  ps <- read_rds(paste0(params$ps_obj_path, i, "__PSobj_original_counts.rds"))
  assign(paste0("ps_", i), ps)
}
```

# Summary of datasets and metadata

## Number of subjects (and their ages) from each dataset included in the analyses

Compute values from phyloseq objects

```
df <- data.frame(dataset = gsub("ps_", "", grep("ps_", ls(), value = T)),
             ASD = unlist(lapply(mget(grep("ps_", ls(), value = T)), function(x) {
               sum(sample_data(x)$Subset_None_Variable_Autism.spectrum.disorder..Biospecimen._Gr_FALSE.over.TRUE == "TRUE", 
                   na.rm = T)}), use.names = F),
             NT = unlist(lapply(mget(grep("ps_", ls(), value = T)), function(x) {
               sum(sample_data(x)$Subset_None_Variable_Autism.spectrum.disorder..Biospecimen._Gr_FALSE.over.TRUE == "FALSE", 
                   na.rm = T)}), use.names = F),
             min_age = unlist(lapply(mget(grep("ps_", ls(), value = T)), function(x) {
               min(sample_data(x)$Subset_None_Variable_Age..years...Biospecimen., na.rm = T)}), use.names = F),
             max_age = unlist(lapply(mget(grep("ps_", ls(), value = T)), function(x) {
               max(sample_data(x)$Subset_None_Variable_Age..years...Biospecimen., na.rm = T)}), use.names = F),
             med_age = unlist(lapply(mget(grep("ps_", ls(), value = T)), function(x) {
               med = median(sample_data(x)$Subset_None_Variable_Age..years...Biospecimen., na.rm = T)
               if (is.null(med)) { med = NA }
               return(med)}), use.names = F),
             male = unlist(lapply(mget(grep("ps_", ls(), value = T)), function(x) {
               sum(sample_data(x)$Subset_None_Variable_Biological.sex..Biospecimen._Gr_Female.over.Male == "Male", 
                   na.rm = T)/length(sample_data(x)$Subset_None_Variable_Biological.sex..Biospecimen._Gr_Female.over.Male)}), use.names = F))
df <- left_join(ASD_datasets[,c("Dataset.ID", "SG.Project.ID", "Platform_group", 
                                "Sequencing.Protocol.instrument_model", "Target_region")], 
                df, by = c("Dataset.ID" = "dataset"))
```

Manually add values reported in publications but not provided as
metadata

```
df$min_age[df$Dataset.ID %in% c("DS4", "DS6")] <- c(2, 2)
df$max_age[df$Dataset.ID %in% c("DS4", "DS6")] <- c(10, 4)
df$ASD[df$Dataset.ID == "DS10"] <- 15
df$NT[df$Dataset.ID == "DS10"] <- 5
df$male[df$Dataset.ID == "DS1"] <- sum(!is.na(ps_DS1@sam_data$Subset_Biological.sex..Biospecimen.__Male_Variable_Autism.spectrum.disorder..Biospecimen._Gr_FALSE.over.TRUE))/nsamples(ps_DS1)
df$male[df$Dataset.ID == "DS3"] <- sum(!is.na(ps_DS3@sam_data$Subset_Biological.sex..Biospecimen.__Male_Variable_Autism.spectrum.disorder..Biospecimen._Gr_FALSE.over.TRUE))/nsamples(ps_DS3)
df$min_age[df$Dataset.ID == "DS5"] <- df$max_age[df$Dataset.ID == "DS5"] <- df$med_age[which(df$Dataset.ID %in% c("DS4", "DS5", "DS6"))] <- df$male[which(df$Dataset.ID %in% c("DS4", "DS5", "DS6"))] <- "not reported"
```

Export for table

```
df <- rename(df, "Dataset" = "Dataset.ID", "Technology" = "Platform_group", 
             "Sequencing instrument" = "Sequencing.Protocol.instrument_model", 
             "16S region" = "Target_region", "ASD (n)" = "ASD", "NT (n)" = "NT", 
             "Minimum age" = "min_age", "Maximum age" = "max_age", "Median age" = "med_age",
             "Male (%)" = "male")
write_csv(df, "dataset_summary_table.csv")
```

### Summary of cohort characteristics by study group

```
df_all <- NULL
for (d in grep("ps_", ls(), value = T)) {
  df <- data.frame(get(d)@sam_data) %>%
    mutate(Dataset = gsub("ps_", "", d)) %>%
    select(starts_with("Subset_None")|starts_with("Dataset")) %>%
    rename_with(function(x) gsub("Subset_None_Variable_", "", x))
  df_all <- bind_rows(df_all, df)
}

df_all <- df_all %>%
  # use largest dataset for each study and remove studies missing these variables
  filter(!Dataset %in% c("DS10", "DS12", "DS13", "DS4", "DS5", "DS6")) %>%
  # need to combine all bowel function variables into one
  unite(Functional.bowel.finding..Biospecimen._Gr_Tends.to.have.abnormal.bowel.function.over.Tends.to.have.normal.bowel.function, 
        Functional.bowel.finding..Biospecimen._Gr_Tends.to.have.constipation.over.Tends.to.have.diarrhea, 
        Functional.bowel.finding..Biospecimen._Gr_Tends.to.have.constipation.over.Tends.to.have.normal.bowel.function,
        col = Bowel_function) %>%
  mutate(Bowel_function = gsub("NA_NA_NA", "Not reported", Bowel_function),
         Bowel_function = str_replace_all(Bowel_function, c("NA" = "", "_" = "")),
         Bowel_function = case_when(grepl("constipation|diarrhea", Bowel_function) ~ "Tends.to.have.abnormal.bowel.function",
                                     !grepl("constipation|diarrhea", Bowel_function) ~ Bowel_function),
         Bowel_function = gsub("\\.", " ", Bowel_function),
         Bowel_function = factor(Bowel_function, levels = c("Tends to have normal bowel function",
                                                            "Tends to have abnormal bowel function",
                                                            "Not reported"))) %>%
  # fix names for plot
  rename("Age" = "Age..years...Biospecimen.",
         "Bowel function" = "Bowel_function",
         "ASD" = "Autism.spectrum.disorder..Biospecimen._Gr_FALSE.over.TRUE") %>%
  mutate(Study = str_replace_all(Dataset, c("DS11" = "Internal",
                                            "DS1" = "Kang 2013",
                                            "DS2" = "Kang 2018",
                                            "DS3" = "Kang 2017/2019",
                                            "DS7" = "Wang 2019",
                                            "DS8" = "Pulikkan 2018",
                                            "DS9" = "Averina 2020")),
         Study = factor(Study, levels = c("Kang 2013", "Kang 2018", "Kang 2017/2019", 
                                          "Wang 2019", "Pulikkan 2018", 
                                            "Averina 2020", "Internal")),
         ASD = factor(str_replace_all(ASD, c("TRUE" = "ASD", "FALSE" = "NT"))))
```

Plot age, sex, GI issues by study group for each cohort

```
p <- ggboxplot(df_all, x = "ASD", y = "Age", color = "black", facet.by = "Study", scales = "free",
               add = "jitter", add.params = list(color = "Bowel function"),
               palette = c(color_pal[1:2], "grey"), xlab = FALSE) +
  rotate_x_text(angle = 45) + 
  theme(axis.text = element_text(size = 7),
        axis.title = element_text(size = 8),
        legend.position = "right",
        strip.text = element_text(size = 8)) +
  stat_compare_means(comparisons = list(c("ASD", "NT")), label = "p.signif", hide.ns = TRUE)
p$layers[[3]]$aes_params$vjust <- 0.65
p$layers[[3]]$aes_params$textsize <- 3
```

```
ggsave(filename = "ages_bowel_dysfunction_study_groups.pdf", plot = p, width = 8, height = 6)
```

### How many individuals remain after subsetting to normal bowel function only?

```
to_plot <- df_all %>%
  filter(Study %in% c("Internal", "Kang 2013", "Wang 2019")) %>%
  group_by(Study, ASD) %>%
  summarise(Total = n()-sum(`Bowel function` == "Tends to have normal bowel function"),
            'Normal bowel' = sum(`Bowel function` == "Tends to have normal bowel function")) %>%
  pivot_longer(cols = c("Total", "Normal bowel"), values_to = "Participants (n)", names_to = "Subsets") %>%
  mutate(Subsets = factor(Subsets, levels = c("Total", "Normal bowel")))
```

```
p <- ggbarplot(to_plot, x = "ASD", y = "Participants (n)", fill = "Subsets", 
               facet.by = "Study", palette = color_pal, xlab = FALSE) + 
  theme(axis.text = element_text(size = 7),
        axis.title = element_text(size = 8))
```

```
ggsave(filename = "normal_bowel_study_groups.pdf", plot = p, height = 4)
```

## Common metadata variables across datasets (upset plot)

Make table to count presence/absence of each variable in each
dataset

```
df <- adonis_res %>%
  mutate(Variable1 = str_replace_all(Test, c("Subset_" = "", "__.*" = "", "_Variable_.*" = "")),
         Variable2 = str_replace_all(Test, c(".*_Variable_" = "", "_Gr_.*" = ""))) %>%
  gather(key = "key", value = "Variable", Variable1, Variable2) %>%
  filter(Variable != "None") %>%
  left_join(ASD_datasets[,c("Dataset", "Dataset.ID")]) %>%
  dplyr::group_by(Dataset.ID, Variable) %>%
  summarise(count = 1) %>%
  spread(key = "Dataset.ID", value = "count", fill = 0) %>%
  data.frame()
```

Upset plot

```
png(file = "ASD_CIMA_vars_upset.png", width = 6, height = 4, units = "in", res = 600)
upset(df, sets = make.names(ASD_datasets$Dataset.ID), keep.order = F, text.scale = 0.6, point.size = 2)
dev.off()
```

```
## quartz_off_screen 
##                 2
```

Supplementary table

```
df$Total <- rowSums(df[,-1])
df$Variable <- str_replace_all(df$Variable, c("\\.\\.Biospecimen\\." = "", "\\.\\." = " - ", "\\." = " "))
write_csv(arrange(df, desc(Total)), "variable_coverage.csv")
```

## Number of tests after variable combinations

Starting number of variables (52) -> number of contrasts ->
expansion to combinations

```
comparisons_sub <- comparisons_sub %>%
  unite(col = combo, Subset, Variable, Group1, Group2, sep = "__", remove = FALSE) %>%
  unite(col = contrast, Variable, Group1, Group2, sep = "__", remove = FALSE)
n_distinct(comparisons_sub$contrast)
```

```
## [1] 70
```

```
n_distinct(comparisons_sub$combo)
```

```
## [1] 580
```

## Percentage of strain hits in each 16S dataset

```
nstrains <- lapply(mget(paste0("ps_DS", c(1:6,8,10:12))), function(x) {
  sum(grepl("t__[[:digit:]]", x@tax_table[,"Strain"]))
})
ntaxa <- lapply(mget(paste0("ps_DS", c(1:6,8,10:12))), function(x) {
  ntaxa(x)
})
# ASVs
round(((nstrains$ps_DS3 + nstrains$ps_DS11)/(ntaxa$ps_DS3 + ntaxa$ps_DS11))*100, digits = 2)
```

```
## [1] 6
```

```
# OTUs
round(((nstrains$ps_DS1 + nstrains$ps_DS2 + nstrains$ps_DS4 + nstrains$ps_DS5 + nstrains$ps_DS6 + nstrains$ps_DS8 + nstrains$ps_DS10)/(ntaxa$ps_DS1 + ntaxa$ps_DS2 + ntaxa$ps_DS4 + ntaxa$ps_DS5 + ntaxa$ps_DS6 + ntaxa$ps_DS8 + ntaxa$ps_DS10))*100, digits = 2)
```

```
## [1] 0.9
```

```
# eOTUs
round((nstrains$ps_DS12/ntaxa$ps_DS12)*100, digits = 2)
```

```
## [1] 6.76
```

# Summary of Adonis results

For each variable which had significant Adonis results in at least 2
datasets, plot the total number of datasets that variable was tested in
and the proportion of datasets with a significant result. Plot for each
taxonomic rank.

```
to_plot <- adonis_res %>%
  mutate(Rank = factor(Rank, levels = c("Phylum", "Class", "Order", "Family", "Genus", "Species", "No agglomeration")),
         Test = gsub("\\.\\.Biospecimen\\.", "", Test)) %>%
  group_by(Test, Rank) %>%
  summarise(Significant = sum(Adonis_p_val < 0.05),
            Total_real = n(),
            Total = n() - Significant)
# keep only variables with significant results in more than one dataset
keep <- unique(to_plot[to_plot$Significant > 1,]$Test)

to_plot <- to_plot %>%
  filter(Test %in% keep) %>%
  gather(key = "Label", value = "number", Significant, Total) %>%
  arrange(desc(Total_real), desc(number)) %>%
  ungroup() %>%
  mutate(Label = factor(Label, levels = c("Total", "Significant")),
         Test = as_factor(Test))

p <- ggbarplot(to_plot, x = "Test", y = "number", fill = "Label", color = FALSE,
               facet.by = "Rank", 
               palette = c("grey", "red"),
               ylab = "Datasets (n)", xlab = "Variable combination") + 
  theme(axis.text.x = element_blank(),
        axis.text.y = element_text(size = 8),
        axis.ticks.x = element_blank(),
        legend.title = element_blank()) +
  scale_y_continuous(breaks = c(1,3,5,7,9)) +
  geom_hline(yintercept = 2, linetype = 3, size = 0.5)
```

```
ggsave(filename = "adonis_overall_summary.pdf", plot = p, width = 7, height = 4)
```

Supplementary table

```
supp_tab <- to_plot %>%
  filter(Label != "Total") %>%
  select(-Label) %>%
  unite(col = "Significant/Total", number, Total_real, sep = " of ", remove = TRUE) %>%
  arrange(Test, Rank) %>%
  spread(key = "Rank", value = "Significant/Total", fill = "NS") %>%
  mutate(Test = str_replace_all(Test, c("Subset_" = "Subset: ",
                                        "_Variable_" = "; Variable: ",
                                        "_Gr_" = " - ",
                                        "__" = " = ",
                                        "\\.\\." = " (",
                                        "\\.$" = ")",
                                        "\\." = " ")))
```

```
write_csv(supp_tab, "adonis_summary_table.csv")
```

## Significance by variable combinations

Heatmaps showing the proportion of datasets that had significant
results for each variable combination at each taxonomic rank

```
for (tax_rank in unique(adonis_res$Rank)) {
  
  df <- adonis_res %>%
    filter(Rank == tax_rank) %>%
    group_by(Test) %>%
    dplyr::summarise(sig = sum(Adonis_p_val < 0.05),
                     total = n(),
                     perc_sig = sig/total) %>%
    filter(total > 1) %>%
    mutate(Subset = str_replace_all(Test, c("Subset_" = "", "_Variable_.*" = "", "\\.\\.Biospecimen\\." = "")),
           Variable = str_replace_all(Test, c(".*_Variable_" = "", "\\.\\.Biospecimen\\." = "")))
  
  # order heatmap
  coln <- names(sort(table(df$Subset), decreasing = TRUE))
  rown <- names(sort(table(df$Variable), decreasing = TRUE))
  df <- df %>%
    mutate(Subset = factor(Subset, levels = coln),
           Variable = factor(Variable, levels = rown)) %>%
    reshape2::dcast(Variable ~ Subset, value.var = "perc_sig", fill = -0.15)
  
  to_plot <- df[,-1]
  row.names(to_plot) <- df$Variable
  
  mat_breaks <- c(-0.15, seq(from = -0.01, to = 1, length.out = 9))
  
  col_pal <- c("lemonchiffon", RColorBrewer::brewer.pal(n = 9, name = "PuBuGn"))
  
  pheatmap::pheatmap(to_plot,
                     cluster_rows = F,
                     cluster_cols = F,
                     treeheight_row = 0,
                     treeheight_col = 0,
                     cellwidth = 8,
                     cellheight = 8,
                     fontsize = 8,
                     breaks = mat_breaks,
                     legend_breaks = mat_breaks,
                     legend_labels = c("Not tested", "0", round(mat_breaks, digits = 2)[-c(1:2)]),
                     color = col_pal,
                     filename = paste0("adonis_heatmap_summary_", tax_rank, ".pdf"))
}
```

# ASD-associated taxa

Which taxa are significant between ASD and NT without subsetting the
data?

```
ma_res_ASD <- ma_res %>%
  filter(CIMA_test == "Subset_None_Variable_Autism.spectrum.disorder..Biospecimen._Gr_FALSE.over.TRUE") %>%
  mutate(p.plot = -log10(Padjust),
         Rank = gsub("NoAgglomeration", "Strain", Rank),
         Rank = factor(Rank, levels = c("Phylum", "Class", "Order", "Family", "Genus", "Species", "Strain")),
         Significance = ifelse(Padjust < 0.05, "q<0.05", "q>0.05"),
         Significance = factor(Significance, levels = c("q>0.05", "q<0.05")),
         label = ifelse(Padjust < 0.05, Bin, NA))
p1 <- ggscatter(ma_res_ASD, x = "Effect_size", y = "p.plot", color = "Rank", size = "Detection_count", alpha = "Significance",
               ylab = "-log10(q)", xlab = "Effect size", label = "label", repel = TRUE, font.label = c(6, "italic", "black"),
               palette = color_pal) +
  geom_vline(xintercept = 0, linetype = 2, color = "grey") +
  geom_hline(yintercept = -log10(0.05), linetype = 2, color = "grey") +
  theme(legend.text = element_text(size = 6),
        legend.title = element_text(size = 7),
        legend.key.size = unit(3, "mm"),
        legend.spacing = unit(3, "mm"),
        axis.text = element_text(size = 7),
        axis.title = element_text(size = 8)) +
  labs(size = "Datasets (n)")
```

```
ggsave(filename = "meta_analysis_ASDvNT.pdf", plot = p1, width = 7, height = 5)
```

Supplementary table

```
df <- ma_res_ASD %>%
  filter(Significance == "q<0.05" & Rank == "NoAgglomeration") %>%
  group_by(Detection_count, Model, Effect_size, Standard_error, Pvalue, 
           CILB, CIUB, CIMA_test, Datasets, hat_values, Padjust) %>%
  dplyr::summarise(Bin = paste(unique(Bin), collapse = " ; "),
                   genus = paste(unique(genus), collapse = " ; "),
                   species = paste(unique(species), collapse = " ; "))
```

```
write_csv(df, "Table_Case_Control_meta_analysis_NoAgglom.csv")
```

## After subsetting

Compare Case vs Control in all children with Case vs Control in only
children with normal bowel function. After removing children with bowel
dysfunction, the taxa associated with ASD or NT are insignificant but
new asscociations are revealed.

```
# keep taxa significant in either model
ma_res_bf <- ma_res %>%
  filter(CIMA_test %in% c("Subset_Functional.bowel.finding..Biospecimen.__Tends.to.have.normal.bowel.function_Variable_Autism.spectrum.disorder..Biospecimen._Gr_FALSE.over.TRUE", "Subset_None_Variable_Autism.spectrum.disorder..Biospecimen._Gr_FALSE.over.TRUE")) %>%
  mutate(CIMA_test = str_replace_all(CIMA_test, c("Subset_Functional.*" = "Normal bowel function only", "Subset_None.*" = "All")))
keep <- ma_res_bf$Bin[ma_res_bf$Padjust < 0.05]

ma_res_bf <- ma_res_bf %>%
  filter(Bin %in% keep) %>%
  # collapse multi-hits
  group_by(Detection_count, Model, Effect_size, Standard_error, Pvalue, 
           CILB, CIUB, CIMA_test, Datasets, hat_values, Padjust) %>%
  dplyr::summarise(Bin = paste(unique(Bin), collapse = " ; "),
                   phylum = paste(gsub("p__", "", unique(phylum)), collapse = " ; "),
                   genus = paste(unique(genus), collapse = " ; "),
                   species = paste(unique(species), collapse = " ; ")) %>%
  # only significant multi-hits are from g_Klebsiella
  mutate(Bin = ifelse(grepl(";", Bin), "unclassified Klebsiella ASV", Bin),
         Significance = ifelse(Padjust < 0.05, "q<0.05", "q>0.05"),
         Significance = factor(Significance, levels = c("q>0.05", "q<0.05"))) %>%
  arrange(Effect_size)
ma_res_bf$Bin[grepl("unclassified Klebsiella", ma_res_bf$Bin)] <- paste0("unclassified Klebsiella ASV", 1:3)
# order plot
ma_res_bf$Bin <- as_factor(ma_res_bf$Bin)
```

Forest plot

```
forest <- ggplot(ma_res_bf, aes(y = Effect_size, x = Bin, color = Detection_count, shape = Significance)) +
  facet_grid(as.formula("phylum~CIMA_test"), scales = "free", space = "free") + 
  geom_errorbar(aes(ymax = CIUB, ymin = CILB), color = "black", width = 0.3) +
  geom_point(size = 2) + 
  geom_hline(yintercept = 0.0, linetype = 2, alpha = 0.75) +
  theme(text = element_text(size = 8)) +
  labs(color = "Datasets (n)",
       y = "Effect size",
       x = "") +
  coord_flip() +
  scale_color_viridis()
```

```
ggsave(filename = "meta_analysis_ASDvNT_normal_bowel.pdf", plot = forest, width = 6, height = 6)
```

### Add individual effect sizes for selected strains

Two strains: t\_\_190463 (Bacteroides stercoris strain, depleted in
ASD), t\_\_180407 (Clostridium M bolteae strain, enriched in ASD) and two
species: Massilioclostridium coli (species, depleted in ASD),
Granulicatella elegans (species, depleted in ASD), are significant only
after removing children with abnormal bowel function. We want to see the
individual effect sizes along with the random-effects models.

```
ma_res_bf_hold <- filter(ma_res_bf, 
                    Bin %in% c("t__190463", "t__180407", "s__Granulicatella__elegans", "s__Massilioclostridium__coli"),
                    CIMA_test == "Normal bowel function only") %>%
  mutate(Type = "Meta-analysis") %>%
  dplyr::select(Bin, Datasets, Effect_size, CILB, CIUB, hat_values, Type)
# get hat values
hold <- as.numeric(unlist(str_split(string = ma_res_bf_hold$hat_values, pattern= " ; ")))
hat_vals <- data.frame(Dataset = unlist(str_split(string = ma_res_bf_hold$Datasets, pattern= "; ")),
                       Bin = rep(ma_res_bf_hold$Bin, times = ma_res_bf_hold$Detection_count),
                       hat_values = hold[!is.na(hold)])

# add individual effect sizes
df <- filter(fs_res,
             CIMA_test == "Subset_Functional.bowel.finding..Biospecimen.__Tends.to.have.normal.bowel.function_Variable_Autism.spectrum.disorder..Biospecimen._Gr_FALSE.over.TRUE",
             bin %in% c("t__190463", "t__180407", "s__Granulicatella__elegans", "s__Massilioclostridium__coli"))
df <- bind_rows(ma_res_bf_hold %>%
                  mutate(hat_values = NA,
                         Dataset_ID = "Meta-analysis"), 
                data.frame(Bin = df$bin, 
                           Dataset = df$Dataset,
                           Dataset_ID = mapvalues(df$Dataset, ASD_datasets$Dataset, ASD_datasets$Dataset.ID),
                           Effect_size = df$EffectSize, 
                           CILB = df$EffectSize-1.95*df$SE,
                           CIUB = df$EffectSize+1.95*df$SE, 
                           Type = "Individual") %>%
                  left_join(hat_vals))
```

Forest plot

```
forest <- ggplot(df, aes(y = Effect_size, x = Dataset_ID, color = hat_values, shape = Type)) +
  facet_wrap(as.formula("~Bin"), scales = "free") + 
  geom_errorbar(aes(ymax = CIUB, ymin = CILB), color = "black", width = 0.3) +
  geom_point(size = 2) + 
  geom_hline(yintercept = 0.0, linetype = 2, alpha = 0.75) +
  theme(text = element_text(size = 8)) +
  labs(y = "Effect size",
       x = "",
       color = "Hat value") +
  coord_flip()
```

```
ggsave(filename = "normal_bowel_ASDvNT_individualES.pdf", plot = forest, width = 6, height = 4)
```

### Prevalence of ASD-associated taxa in children with normal bowel function

```
sel_tax <- ma_res_bf %>%
  filter(CIMA_test == "Normal bowel function only",
         Significance == "q<0.05",
         abs(Effect_size) > 2)
```

Get relative abundances from 4 datasets

```
ASD_datasets$Dataset.ID[ASD_datasets$Dataset %in% unique(unlist(str_split(sel_tax$Datasets, pattern = "; ")))]
```

```
## [1] "DS1"  "DS7"  "DS11" "DS13"
```

```
# DS1 - "ASU_RKrajmalnikBrown_BIRD13_0286-16S sequencing-Stool specimen-DNA"
hold_asv <- sg_normalize(ps_DS1, method = "tss")
hold_sp <- sg_normalize(tax_glom(ps_DS1, taxrank = "Species"), method = "tss")
DS1_RA <- data.frame(ASD = ps_DS1@sam_data$Subset_Functional.bowel.finding..Biospecimen.__Tends.to.have.normal.bowel.function_Variable_Autism.spectrum.disorder..Biospecimen._Gr_FALSE.over.TRUE,
                     Dataset = "DS1",
                     t__180407 = 0, # not present in this dataset
                     t__190463 = as(hold_asv@otu_table, "matrix")["dnOTU_17",],
                     s__Granulicatella__elegans = as(hold_sp@otu_table, "matrix")[which(hold_sp@tax_table[,"Species"] == "s__Granulicatella__elegans"),],
                     s__Massilioclostridium__coli = as(hold_sp@otu_table, "matrix")[which(hold_sp@tax_table[,"Species"] == "s__Massilioclostridium__coli"),])

# DS7 - "CHFU_WZhou_BIRD19_0291-Metagenome-Stool specimen-DNA"
hold_asv <- ps_DS7
DS7_RA <- data.frame(ASD = ps_DS7@sam_data$Subset_Functional.bowel.finding..Biospecimen.__Tends.to.have.normal.bowel.function_Variable_Autism.spectrum.disorder..Biospecimen._Gr_FALSE.over.TRUE,
                     Dataset = "DS7",
                     t__180407 = as(hold_asv@otu_table, "matrix")["t__180407",],
                     t__190463 = as(hold_asv@otu_table, "matrix")["t__190463",],
                     s__Granulicatella__elegans = 0,
                     s__Massilioclostridium__coli = 0)

# DS11 - "SG_SIwai_INRD17_0415-16S sequencing-Stool specimen-DNA"
hold_asv <- sg_normalize(ps_DS11, method = "tss")
hold_sp <- sg_normalize(tax_glom(ps_DS11, taxrank = "Species"), method = "tss")
DS11_RA <- data.frame(ASD = ps_DS11@sam_data$Subset_Functional.bowel.finding..Biospecimen.__Tends.to.have.normal.bowel.function_Variable_Autism.spectrum.disorder..Biospecimen._Gr_FALSE.over.TRUE,
                      Dataset = "DS11",
                      t__180407 = as(hold_asv@otu_table, "matrix")["GCAAGCGTTATCCGGATTTACTGGGTGTAAAGGGAGCGTAGACGGCGAAGCAAGTCTGAAGTGAAAACCCAGGGCTCAACCCTGGGACTGCTTTGGAAACTGTTTTGCTAGAGTGTCGGAGAGGTAAGTGGAATTCCTAGTGTAGCGGTGAAATGCGTAGATATTAGGAGGAACACCAGTGGCGAAGGCGGCTTACTGGACGATAACTGACGTTGAGGCTCGAAAGCGTGGGG",],
                      t__190463 = as(hold_asv@otu_table, "matrix")["CCGAGCGTTATCCGGATTTATTGGGTTTAAAGGGAGCGTAGGCGGGTTGTTAAGTCAGTTGTGAAAGTTTGCGGCTCAACCGTAAAATTGCAGTTGATACTGGCGACCTTGAGTGCAACAGAGGTAGGCGGAATTCGTGGTGTAGCGGTGAAATGCTTAGATATCACGAAGAACTCCGATTGCGAAGGCAGCTTACTGGATTGTAACTGACGCTGATGCTCGAAAGTGTGGGT",],
                      s__Granulicatella__elegans = as(hold_sp@otu_table, "matrix")[which(hold_sp@tax_table[,"Species"] == "s__Granulicatella__elegans"),],
                      s__Massilioclostridium__coli = as(hold_sp@otu_table, "matrix")[which(hold_sp@tax_table[,"Species"] == "s__Massilioclostridium__coli"),])

# DS13 - "SG_SIwai_INRD17_0415-Metagenome-Stool specimen-DNA"
hold_asv <- ps_DS13
DS13_RA <- data.frame(ASD = ps_DS13@sam_data$Subset_Functional.bowel.finding..Biospecimen.__Tends.to.have.normal.bowel.function_Variable_Autism.spectrum.disorder..Biospecimen._Gr_FALSE.over.TRUE,
                      Dataset = "DS13",
                      t__180407 = as(hold_asv@otu_table, "matrix")["t__180407",],
                      t__190463 = as(hold_asv@otu_table, "matrix")["t__190463",],
                      s__Granulicatella__elegans = 0,
                      s__Massilioclostridium__coli = 0)

to_plot <- rbind(DS1_RA, DS7_RA, DS11_RA, DS13_RA) %>%
  filter(!is.na(ASD)) %>% # keeping only kids with normal bowel function
  pivot_longer(cols = c("t__180407", "t__190463", "s__Granulicatella__elegans", "s__Massilioclostridium__coli"),
               names_to = "Taxon", values_to = "Relative_abundance") %>%
  mutate(Relative_abundance = as.numeric(gsub(NaN, 0, Relative_abundance)))
```

Prevalence of selected taxa (non-zero abundance)

```
table(subset(to_plot, to_plot$Relative_abundance > 0)$ASD, subset(to_plot, to_plot$Relative_abundance != 0)$Taxon)
```

```
##        
##         s__Granulicatella__elegans s__Massilioclostridium__coli t__180407
##   FALSE                          5                           12        68
##   TRUE                           0                            0        50
##        
##         t__190463
##   FALSE        53
##   TRUE         32
```

The two species have very low prevalence (G. elegans prevalence =
0.0308642; M. coli prevalence = 0.0740741). Plot the strains (t\_\_180407
prevalence = 0.3794212; t\_\_190463 prevalence = 0.2478134)

### Within ASD group differences by bowel function

ASD children with diarrhea have a different taxonomic profile
compared to ASD children with constipation.

```
ma_res_ASD_GI <- ma_res %>%
  filter(CIMA_test %in% c("Subset_Autism.spectrum.disorder..Biospecimen.__TRUE_Variable_Functional.bowel.finding..Biospecimen._Gr_Tends.to.have.constipation.over.Tends.to.have.diarrhea")) %>%
  mutate(p.plot = -log10(Padjust),
         Rank = gsub("NoAgglomeration", "Strain", Rank),
         Rank = factor(Rank, levels = c("Phylum", "Class", "Order", "Family", "Genus", "Species", "Strain")),
         Significance = ifelse(Padjust < 0.05, "q<0.05", "q>0.05"),
         Significance = factor(Significance, levels = c("q>0.05", "q<0.05")),
         label = ifelse(Padjust < 0.05 & !grepl("^t__", Bin), Bin, NA))

p1 <- ggscatter(ma_res_ASD_GI, x = "Effect_size", y = "p.plot", color = "Rank", size = "Detection_count", alpha = "Significance",
               ylab = "-log10(q)", xlab = "Effect size", label = "label", repel = TRUE, font.label = c(6, "italic", "black"),
               palette = color_pal) +
  geom_vline(xintercept = 0, linetype = 2, color = "grey") +
  geom_hline(yintercept = -log10(0.05), linetype = 2, color = "grey") +
  theme(legend.text = element_text(size = 6),
        legend.title = element_text(size = 7),
        legend.key.size = unit(3, "mm"),
        legend.spacing = unit(3, "mm"),
        axis.text = element_text(size = 7),
        axis.title = element_text(size = 8)) + 
  scale_size(breaks = c(2:4), range = c(2,4)) +
  labs(size = "Datasets (n)")
```

```
ggsave(filename = "meta_analysis_ASD_bowel_function.pdf", plot = p1, width = 7, height = 5)
```

Supplementary table

```
df <- ma_res_ASD_GI %>%
  filter(Significance == "q<0.05" & Rank == "NoAgglomeration") %>%
  group_by(Detection_count, Model, Effect_size, Standard_error, Pvalue, 
           CILB, CIUB, CIMA_test, Datasets, hat_values, Padjust) %>%
  dplyr::summarise(Bin = paste(unique(Bin), collapse = " ; "),
                   genus = paste(unique(genus), collapse = " ; "),
                   species = paste(unique(species), collapse = " ; "))
```

```
write_csv(df, "Table_ASD_bowel_function_meta_analysis_NoAgglom.csv")
```

### Childhood versus adolescence

Taxa which discriminate between ASD and NT children are different in
young children vs adolescents.

```
ma_res_ASD <- ma_res %>%
  filter(CIMA_test %in% c("Subset_Period.of.life..Biospecimen.__Childhood_Variable_Autism.spectrum.disorder..Biospecimen._Gr_FALSE.over.TRUE",
                          "Subset_Period.of.life..Biospecimen.__Adolescence_Variable_Autism.spectrum.disorder..Biospecimen._Gr_FALSE.over.TRUE")) %>%
  mutate(CIMA_test = ifelse(grepl("Childhood", CIMA_test), "Childhood - NT over ASD", "Adolescence - NT over ASD"),
         CIMA_test = factor(CIMA_test, levels = rev(unique(CIMA_test))),
         p.plot = -log10(Padjust),
         Rank = gsub("NoAgglomeration", "Strain", Rank),
         Rank = factor(Rank, levels = c("Phylum", "Class", "Order", "Family", "Genus", "Species", "Strain")),
         Significance = ifelse(Padjust < 0.05, "q<0.05", "q>0.05"),
         Significance = factor(Significance, levels = c("q>0.05", "q<0.05")),
         label = ifelse(Padjust < 0.05 & !grepl("^t__", Bin), Bin, NA))

p1 <- ggscatter(ma_res_ASD, x = "Effect_size", y = "p.plot", color = "Rank", size = "Detection_count", alpha = "Significance",
               ylab = "-log10(q)", xlab = "Effect size", label = "label", repel = TRUE, font.label = c(6, "italic", "black"),
               facet = "CIMA_test", palette = color_pal) +
  geom_vline(xintercept = 0, linetype = 2, color = "grey") +
  geom_hline(yintercept = -log10(0.05), linetype = 2, color = "grey") +
  theme(legend.text = element_text(size = 6),
        legend.title = element_text(size = 7),
        legend.key.size = unit(3, "mm"),
        legend.spacing = unit(3, "mm"),
        axis.text = element_text(size = 7),
        axis.title = element_text(size = 8)) + 
  ylim(0,11) + 
  xlim(c(-15,15)) +
  labs(size = "Datasets (n)")
```

```
ggsave(filename = "meta_analysis_ASDvNT_subsets.pdf", plot = p1, width = 7.5, height = 6.5)
```

Supplementary table

```
df <- ma_res_ASD %>%
  filter(Significance == "q<0.05" & Rank == "NoAgglomeration") %>%
  group_by(Detection_count, Model, Effect_size, Standard_error, Pvalue, 
           CILB, CIUB, CIMA_test, Datasets, hat_values, Padjust) %>%
  dplyr::summarise(Bin = paste(unique(Bin), collapse = " ; "),
                   genus = paste(unique(genus), collapse = " ; "),
                   species = paste(unique(species), collapse = " ; "))
```

```
write_csv(df, "Table_Case_Control_meta_analysis_NoAgglom_subsets.csv")
```

### Male vs Female

Gender differences in the gut microbiota are stronger in the ASD
population than the NT population.

```
ma_res_MF1 <- ma_res %>%
  filter(CIMA_test %in% c("Subset_Autism.spectrum.disorder..Biospecimen.__FALSE_Variable_Biological.sex..Biospecimen._Gr_Female.over.Male",
                          "Subset_Autism.spectrum.disorder..Biospecimen.__TRUE_Variable_Biological.sex..Biospecimen._Gr_Female.over.Male")) %>%
  mutate(CIMA_test = ifelse(grepl("TRUE", CIMA_test), "ASD - Female over Male", "NT - Female over Male"),
         p.plot = -log10(Padjust),
         Rank = gsub("NoAgglomeration", "Strain", Rank),
         Rank = factor(Rank, levels = c("Phylum", "Class", "Order", "Family", "Genus", "Species", "Strain")),
         Significance = ifelse(Padjust < 0.05, "q<0.05", "q>0.05"),
         Significance = factor(Significance, levels = c("q>0.05", "q<0.05")),
         label = ifelse(Padjust < 0.05 & !grepl("^t__", Bin), Bin, NA))

ma_res_MF2 <- ma_res %>%
  filter(CIMA_test %in% c("Subset_Biological.sex..Biospecimen.__Male_Variable_Autism.spectrum.disorder..Biospecimen._Gr_FALSE.over.TRUE",
                          "Subset_Biological.sex..Biospecimen.__Female_Variable_Autism.spectrum.disorder..Biospecimen._Gr_FALSE.over.TRUE")) %>%
  mutate(CIMA_test = ifelse(grepl("Male", CIMA_test), "Male - NT over ASD", "Female - NT over ASD"),
         p.plot = -log10(Padjust),
         Rank = gsub("NoAgglomeration", "Strain", Rank),
         Rank = factor(Rank, levels = c("Phylum", "Class", "Order", "Family", "Genus", "Species", "Strain")),
         Significance = ifelse(Padjust < 0.05, "q<0.05", "q>0.05"),
         Significance = factor(Significance, levels = c("q>0.05", "q<0.05")),
         label = ifelse(Padjust < 0.05 & !grepl("^t__", Bin), Bin, NA))
```

```
p1 <- ggscatter(ma_res_MF1, x = "Effect_size", y = "p.plot", color = "Rank", size = "Detection_count", alpha = "Significance",
               ylab = "-log10(q)", xlab = "Effect size", label = "label", repel = TRUE, font.label = c(5, "italic", "black"),
               facet = "CIMA_test", palette = color_pal) +
  geom_vline(xintercept = 0, linetype = 2, color = "grey") +
  geom_hline(yintercept = -log10(0.05), linetype = 2, color = "grey") +
  theme(legend.text = element_text(size = 6),
        legend.title = element_text(size = 7),
        legend.key.size = unit(3, "mm"),
        legend.spacing = unit(3, "mm"),
        axis.text = element_text(size = 7),
        axis.title = element_text(size = 8)) + 
  ylim(0,11) + 
  xlim(c(-15,15)) +
  labs(size = "Datasets (n)")

p2 <- ggscatter(ma_res_MF2, x = "Effect_size", y = "p.plot", color = "Rank", size = "Detection_count", alpha = "Significance",
               ylab = "-log10(q)", xlab = "Effect size", label = "label", repel = TRUE, font.label = c(5, "italic", "black"),
               facet = "CIMA_test", palette = color_pal) +
  geom_vline(xintercept = 0, linetype = 2, color = "grey") +
  geom_hline(yintercept = -log10(0.05), linetype = 2, color = "grey") +
  theme(legend.text = element_text(size = 6),
        legend.title = element_text(size = 7),
        legend.key.size = unit(3, "mm"),
        legend.spacing = unit(3, "mm"),
        axis.text = element_text(size = 7),
        axis.title = element_text(size = 8)) + 
  ylim(0,11) + 
  xlim(c(-15,15)) +
  labs(size = "Datasets (n)")
```

```
ggsave(filename = "meta_analysis_maleVSfemale_subsets.pdf", plot = ggarrange(p1, p2, nrow = 2, common.legend = T), 
       width = 8, height = 9)
```

Supplementary table

```
df <- bind_rows(ma_res_MF1, ma_res_MF2) %>%
  filter(Significance == "q<0.05" & Rank == "NoAgglomeration") %>%
  group_by(Detection_count, Model, Effect_size, Standard_error, Pvalue, 
           CILB, CIUB, CIMA_test, Datasets, hat_values, Padjust) %>%
  dplyr::summarise(Bin = paste(unique(Bin), collapse = " ; "),
                   genus = paste(unique(genus), collapse = " ; "),
                   species = paste(unique(species), collapse = " ; ")) %>%
  arrange(species, desc(Detection_count)) %>%
  arrange(CIMA_test)
```

```
write_csv(df, "Table_Male_Female_meta_analysis_NoAgglom_subsets.csv")
```

# ASD-associated taxa after adjusting for confounders

Feature selection (`DESeq2`) including confounders in the
design. Can only be used at strain level and only on 16S datasets. Need
to retrieve the original metadata (without variable combinations).

```
meta <- read_csv(params$rc) %>%
  select(Autism.spectrum.disorder..Biospecimen., Age..years...Biospecimen., Period.of.life..Biospecimen.,
         Functional.bowel.finding..Biospecimen., Biological.sex..Biospecimen., 
         SG.Project.ID, OmicsFileSet.Barcode)
colnames(meta) <- gsub("\\.\\.Biospecimen\\.", "", colnames(meta))
meta_sub <- filter(meta, 
                   !is.na(meta$Age..years.),
                   !is.na(meta$Functional.bowel.finding),
                   !is.na(meta$Biological.sex))
```

Only 2 datasets from 2 studies have all metadata variables
(Biological sex, age, and bowel dysfunction) for ASD and NT. Run feature
selection with confounder adjustment.

```
fs_adj <- NULL
for (ps in c("DS1", 
             #DS3, bowel function only recorded for ASD
             "DS11")) {
  
  ps <- get(paste0("ps_", ps))
  
  # replace sample data with original metadata
  hold_meta <- meta_sub
  if (all(!sample_names(ps) %in% hold_meta$OmicsFileSet.Barcode)) {
    hold_meta$OmicsFileSet.Barcode <- gsub("_.*", "", hold_meta$OmicsFileSet.Barcode)
    hold_meta <- subset(hold_meta, !duplicated(hold_meta$OmicsFileSet.Barcode))
  }
  hold_meta <- subset(hold_meta, hold_meta$OmicsFileSet.Barcode %in% sample_names(ps))
  row.names(hold_meta) <- hold_meta$OmicsFileSet.Barcode
  ps@sam_data <- sample_data(hold_meta)
  # filter prevalence (5%) and low seq depth (1%)
  ps_filt <- filter_phyloseq(ps, "16S_HTS", "No agglomeration", 0.05, 
                             0.01, 0.01, 0.01, NA)
  # run feature selection
  fs <- deseq2_fs_design(ps_filt,
                         "Autism.spectrum.disorder",
                         "Biological.sex + Age..years. + Functional.bowel.finding + Autism.spectrum.disorder",
                         contrastTop = "FALSE",
                         contrastBottom = "TRUE",
                         parallel = FALSE)
  
  fs <- fs %>%
    mutate(Dataset = ps@sam_data$SG.Project.ID[1],
           CIMA_test = "ASD (adjusted for Age, Sex, Bowel function)")
  
  fs_adj <- rbind(fs_adj, fs)
}
```

Compare results from unadjusted models and adjusted models in ASU
study

```
fs_unadj <- fs_res %>%
  filter(Dataset == "ASU_RKrajmalnikBrown_BIRD13_0286-16S sequencing-Stool specimen-DNA",
         CIMA_test == "Subset_None_Variable_Autism.spectrum.disorder..Biospecimen._Gr_FALSE.over.TRUE",
         Bin_type == "Unique-strain hit") %>%
  left_join(ss_tax, by = c("bin" = "StrainSelectID")) 

fs_adj_ASU <- fs_adj %>%
  filter(Dataset == "ASU_RKrajmalnikBrown_BIRD13_0286",
         grepl("t__", bin)) %>%
  select(bin, Effect_size, Standard_error, Pvalue, Padjust) %>%
  rename_all(~paste0(., "_adjusted")) %>%
  left_join(select(ss_tax, StrainSelectID, family, genus, species), by = c("bin_adjusted" = "StrainSelectID")) 
  
fs_adj_ASU <- fs_unadj %>%
  select(Dataset, bin, EffectSize, SE, pvalue, padjust, family, genus, species) %>%
  full_join(fs_adj_ASU, by = c("bin" = "bin_adjusted", "family", "genus", "species")) %>%
  mutate(Dataset = "ASU_RKrajmalnikBrown_BIRD13_0286-16S sequencing-Stool specimen-DNA")

fs_adj_ASU <- subset(fs_adj_ASU, fs_adj_ASU$padjust < 0.05 | fs_adj_ASU$Padjust_adjusted < 0.05) %>%
  arrange(padjust, Padjust_adjusted)
```

```
write_csv(fs_adj_ASU, "adjusted_models_FS_ASU.csv")
```

In M3 dataset without adjustment, no significant features were
observed. With adjustment, significant features were uncovered.

```
fs_unadj <- fs_res %>%
  filter(Dataset == "SG_SIwai_INRD17_0415-16S sequencing-Stool specimen-DNA",
         CIMA_test == "Subset_None_Variable_Autism.spectrum.disorder..Biospecimen._Gr_FALSE.over.TRUE",
         Bin_type == "Unique-strain hit") %>%
  left_join(ss_tax, by = c("bin" = "StrainSelectID"))

fs_adj_M3 <- fs_adj %>%
  filter(Dataset == "SG_SIwai_INRD17_0415",
         grepl("t__", bin)) %>%
  select(bin, Effect_size, Standard_error, Pvalue, Padjust) %>%
  rename_all(~paste0(., "_adjusted")) %>%
  left_join(select(ss_tax, StrainSelectID, family, genus, species), by = c("bin_adjusted" = "StrainSelectID")) 

fs_adj_M3 <- fs_unadj %>%
  select(Dataset, bin, EffectSize, SE, pvalue, padjust, family, genus, species) %>%
  full_join(fs_adj_M3, by = c("bin" = "bin_adjusted", "family", "genus", "species")) %>%
  mutate(Dataset = "SG_SIwai_INRD17_0415-16S sequencing-Stool specimen-DNA")

fs_adj_M3 <- subset(fs_adj_M3, fs_adj_M3$padjust < 0.05 | fs_adj_M3$Padjust_adjusted < 0.05)
```

```
write_csv(fs_adj_M3, "adjusted_models_FS_M3.csv")
```

Plot before/after confounder adjustment

```
to_plot <- data.frame(Dataset = c("DS1", "DS11"), 
                      Unadjusted_model = c(sum(fs_adj_ASU$padjust < 0.05, na.rm = TRUE), sum(fs_adj_M3$padjust < 0.05, na.rm = TRUE)), 
                      Adjusted_model = c(sum(fs_adj_ASU$Padjust_adjusted < 0.05, na.rm = TRUE), sum(fs_adj_M3$Padjust_adjusted < 0.05, na.rm = TRUE))) %>%
  pivot_longer(cols = c("Unadjusted_model", "Adjusted_model"), names_to = "model", values_to = "Significant strains") %>%
  mutate(model = gsub("_", " ", model))
```

Plot

```
p <- ggbarplot(to_plot, x = "model", y = "Significant strains", xlab = FALSE, facet.by = "Dataset", fill = "model",
               palette = color_pal) +
  theme(legend.position = "none",
        axis.text = element_text(size = 8),
        axis.title = element_text(size = 9))
```

```
ggsave(filename = "number_of_significant_strains_before_after_adjust.pdf", plot = p, width = 4.5, height = 3)
```

Session info

```
sessionInfo()
```

```
## R version 4.2.1 (2022-06-23)
## Platform: x86_64-apple-darwin17.0 (64-bit)
## Running under: macOS Big Sur ... 10.16
## 
## Matrix products: default
## BLAS:   /Library/Frameworks/R.framework/Versions/4.2/Resources/lib/libRblas.0.dylib
## LAPACK: /Library/Frameworks/R.framework/Versions/4.2/Resources/lib/libRlapack.dylib
## 
## locale:
## [1] en_US.UTF-8/en_US.UTF-8/en_US.UTF-8/C/en_US.UTF-8/en_US.UTF-8
## 
## attached base packages:
## [1] stats4    stats     graphics  grDevices utils     datasets  methods  
## [8] base     
## 
## other attached packages:
##  [1] forcats_0.5.1               stringr_1.4.0              
##  [3] dplyr_1.0.9                 purrr_0.3.4                
##  [5] readr_2.1.2                 tidyr_1.2.0                
##  [7] tibble_3.1.8                tidyverse_1.3.2            
##  [9] data.table_1.14.2           aws.s3_0.3.21              
## [11] cima_0.2.0                  viridis_0.6.2              
## [13] viridisLite_0.4.0           UpSetR_1.4.0               
## [15] ggpubr_0.4.0                mixOmics_6.20.0            
## [17] lattice_0.20-45             MASS_7.3-58.1              
## [19] plyr_1.8.7                  DESeq2_1.36.0              
## [21] SummarizedExperiment_1.26.1 Biobase_2.56.0             
## [23] MatrixGenerics_1.8.1        matrixStats_0.62.0         
## [25] GenomicRanges_1.48.0        GenomeInfoDb_1.32.3        
## [27] IRanges_2.30.0              S4Vectors_0.34.0           
## [29] BiocGenerics_0.42.0         secondgenomeR_4.2.1        
## [31] pander_0.6.5                knitr_1.39                 
## [33] ggplot2_3.3.6               phyloseq_1.40.0            
## 
## loaded via a namespace (and not attached):
##   [1] utf8_1.2.2             reticulate_1.25        tidyselect_1.1.2      
##   [4] htmlwidgets_1.5.4      RSQLite_2.2.15         AnnotationDbi_1.58.0  
##   [7] grid_4.2.1             dunn.test_1.3.5        BiocParallel_1.30.3   
##  [10] aws.signature_0.6.0    pROC_1.18.0            munsell_0.5.0         
##  [13] ragg_1.2.2             codetools_0.2-18       DT_0.24               
##  [16] future_1.27.0          withr_2.5.0            colorspace_2.0-3      
##  [19] highr_0.9              logger_0.2.2           botor_0.3.0           
##  [22] rstudioapi_0.13        robustbase_0.95-0      bayesm_3.1-4          
##  [25] ggsignif_0.6.3         listenv_0.8.0          labeling_0.4.2        
##  [28] GenomeInfoDbData_1.2.8 pheatmap_1.0.12        farver_2.1.1          
##  [31] bit64_4.0.5            rhdf5_2.40.0           parallelly_1.32.1     
##  [34] vctrs_0.4.1            generics_0.1.3         metafor_3.4-0         
##  [37] ipred_0.9-13           xfun_0.32              R6_2.5.1              
##  [40] locfit_1.5-9.6         bitops_1.0-7           rhdf5filters_1.8.0    
##  [43] cachem_1.0.6           DelayedArray_0.22.0    assertthat_0.2.1      
##  [46] vroom_1.5.7            scales_1.2.0           googlesheets4_1.0.1   
##  [49] nnet_7.3-17            gtable_0.3.0           globals_0.16.0        
##  [52] timeDate_4021.104      rlang_1.0.4            genefilter_1.78.0     
##  [55] systemfonts_1.0.4      splines_4.2.1          rstatix_0.7.0         
##  [58] gargle_1.2.0           ModelMetrics_1.2.2.2   broom_1.0.0           
##  [61] checkmate_2.1.0        modelr_0.1.8           yaml_2.3.5            
##  [64] reshape2_1.4.4         abind_1.4-5            crosstalk_1.2.0       
##  [67] backports_1.4.1        caret_6.0-93           tensorA_0.36.2        
##  [70] tools_4.2.1            lava_1.6.10            RMariaDB_1.2.2        
##  [73] ellipsis_0.3.2         gplots_3.1.3           jquerylib_0.1.4       
##  [76] biomformat_1.24.0      RColorBrewer_1.1-3     ggdendro_0.1.23       
##  [79] Rcpp_1.0.9             base64enc_0.1-3        zlibbioc_1.42.0       
##  [82] RCurl_1.98-1.8         rpart_4.1.16           Wrench_1.14.0         
##  [85] cowplot_1.1.1          haven_2.5.0            ggrepel_0.9.1         
##  [88] cluster_2.1.3          fs_1.5.2               factoextra_1.0.7      
##  [91] magrittr_2.0.3         RSpectra_0.16-1        reprex_2.0.1          
##  [94] googledrive_2.0.0      hms_1.1.1              evaluate_0.16         
##  [97] xtable_1.8-4           XML_3.99-0.10          readxl_1.4.0          
## [100] mclust_5.4.10          gridExtra_2.3          shape_1.4.6           
## [103] compiler_4.2.1         ellipse_0.4.3          KernSmooth_2.23-20    
## [106] crayon_1.5.1           aws.ec2metadata_0.2.0  htmltools_0.5.3       
## [109] tzdb_0.3.0             mgcv_1.8-40            corpcor_1.6.10        
## [112] geneplotter_1.74.0     lubridate_1.8.0        DBI_1.1.3             
## [115] dbplyr_2.2.1           compositions_2.0-4     Matrix_1.4-1          
## [118] ade4_1.7-19            car_3.1-0              permute_0.9-7         
## [121] cli_3.3.0              parallel_4.2.1         gower_1.0.0           
## [124] igraph_1.3.4           pkgconfig_2.0.3        recipes_1.0.1         
## [127] xml2_1.3.3             foreach_1.5.2          rARPACK_0.11-0        
## [130] annotate_1.74.0        bslib_0.4.0            hardhat_1.2.0         
## [133] multtest_2.52.0        XVector_0.36.0         prodlim_2019.11.13    
## [136] rvest_1.0.2            digest_0.6.29          vegan_2.6-2           
## [139] Biostrings_2.64.0      cellranger_1.1.0       rmarkdown_2.14        
## [142] edgeR_3.38.4           curl_4.3.2             gtools_3.9.3          
## [145] lifecycle_1.0.1        nlme_3.1-159           jsonlite_1.8.0        
## [148] Rhdf5lib_1.18.2        carData_3.0-5          limma_3.52.2          
## [151] fansi_1.0.3            pillar_1.8.0           KEGGREST_1.36.3       
## [154] fastmap_1.1.0          httr_1.4.3             DEoptimR_1.0-11       
## [157] survival_3.4-0         glue_1.6.2             metadat_1.2-0         
## [160] png_0.1-7              iterators_1.0.14       glmnet_4.1-4          
## [163] bit_4.0.4              class_7.3-20           stringi_1.7.8         
## [166] sass_0.4.2             metagenomeSeq_1.38.0   blob_1.2.3            
## [169] textshaping_0.3.6      caTools_1.18.2         memoise_2.0.1         
## [172] mathjaxr_1.6-0         future.apply_1.9.0     ape_5.6-2
```
